# GDNF promotes hair formation and cutaneous wound healing by targeting bulge stem cells

**DOI:** 10.1038/s41536-020-0098-z

**Published:** 2020-06-12

**Authors:** Thomas S. Lisse, Manju Sharma, Neda Vishlaghi, Sri Ramulu Pullagura, Robert E. Braun

**Affiliations:** 1 The Jackson Laboratory, 600 Main Street, Bar Harbor, ME 04609 USA; 2 Department of Biology, The University of Miami, 1301 Memorial Drive, Cox Science Building, Coral Gables, FL 33124 USA; 3 Sylvester Comprehensive Cancer Center, Miller School of Medicine, University of Miami, Miami, FL 33146 USA; 4 Graduate School of Biomedical Sciences and Engineering, University of Maine, Orono, 04469 ME USA

**Keywords:** Skin stem cells, Stem-cell niche

## Abstract

Glial-cell-derived neurotrophic factor (GDNF) is a well-studied neuroregenerative factor; however, the degree to which it supports hair formation and skin wound repair is not known. By using a *Gfra1* (GDNF family receptor alpha 1) knock-in reporter mouse line, GDNF signaling was found to occur within hair bulge stem cells (BSCs) during the initiation of the hair cycle and early stages of hair formation after depilation. Both recombinant and transgene overexpression of GDNF promoted BSC colony growth, hair formation, and skin repair after wounding through enhanced self-renewal of BSCs and commitment of BSC-derived progenitors into becoming epidermal cells at the injury site. Conditional ablation of *Gfra1* among BSCs impaired the onset of the hair cycle, while conditional ablation of the GDNF family member signal transducer, *Ret*, within BSCs prevented the onset of the hair cycle and depilation-induced anagen development of hair follicles. Our findings reveal that GDNF promotes hair formation and wound repair and that bulge stem cells are critical mediators of both.

## Introduction

Hair follicles (HFs) are formed through complex epithelial-mesenchymal interactions. In mice, the morphogenic period of hair formation ends at approximately postnatal day 14 (P14) when HFs establish a definitive bulge stem cell (BSC) compartment^1–3^. BSCs are quiescent and serve as a long-lived pool for regeneration of the lower portion of HFs during cycle activation and injury^4–6^. BSCs retain methylated histones^7^, and express several distinct molecular markers, namely CD34 and keratin 15 (K15)^8^. During the post-morphogenic period, the lower part of the HF undergoes cyclic transformations of growth, involution, and quiescence. These transitions are termed the anagen, catagen, and telogen stages, respectively^3^. The anagen-to-catagen transition marks the end of the active growth phase by deleting, via apoptosis, the old hair shaft. This positions the remaining dermal papilla (Dp), which serves as an inductive signaling center, within close proximity to BSCs to start a new hair cycle upon activation. BSCs also contribute to the healing of wounded skin. During cutaneous wounding of adult skin, a reservoir of BSCs from pre-exiting follicles and/or the interfollicular epithelium (IFE) display plasticity to either become transiently or permanently committed to the neoepidermis of wounds and HFs^9–15^.

Glial-cell-line-derived neurotrophic factor (GDNF) was first identified and characterized as a potent neuroprotective and axonal regenerative factor for subpopulations of dopaminergic^16^ and motor neurons^17,18^. Subsequently it was found to maintain mammalian spermatogonial stem cell (SSC) self-renewal within the testis^19,20^, and to regulate both renal organogenesis^21^ and enteric neurogenesis^22,23^ in mice. GDNF elicits its biological effects through a complex consisting of the RET proto-oncogene transmembrane receptor tyrosine kinase and a glycosylphosphatidylinositol (GPI)-anchored cell surface co-receptor belonging to the GDNF receptor alpha (GFRA) family^18^. Although there are four GFRA family members, GDNF preferentially binds to GFRA1 to elicit activation of its signaling cascade^24^. Several receptor-protein tyrosine kinases can regulate the hair cycle^25^. The RET tyrosine kinase has been implicated in the induction of melanogenesis to provide pigment for HFs^26^, whereby follicular melanogenesis is coupled to the anagen stage of the hair cycle^27^. Although the *Gfra1* transcript was shown to be enriched in BSCs^28,29^, the functional impact of GDNF signaling within BSCs on the hair cycle, and hair and cutaneous wound healing after injury, is currently unknown. The purpose of this study was to determine if GDNF can promote hair formation and skin wound repair by targeting BSCs.

## Results

### GDNF initiates the anagen stage of the hair cycle to promote hair formation

**Fig. 1:**
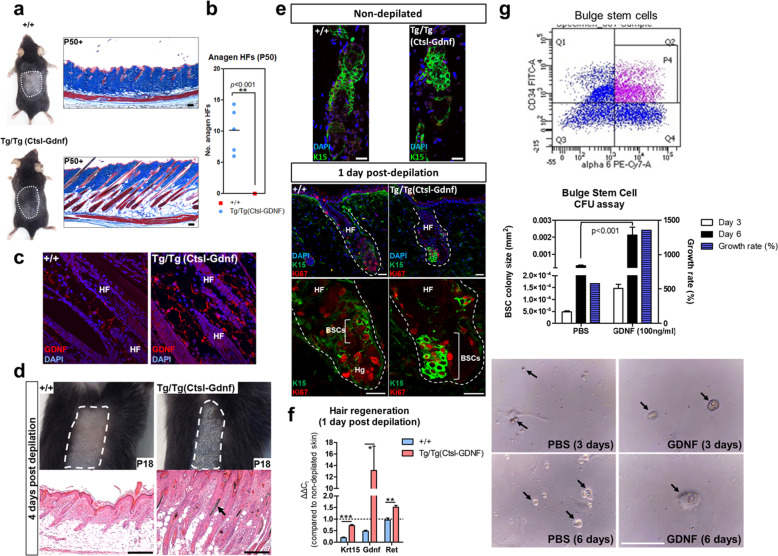
GDNF promotes hair formation. **a** GDNF overexpression in transgenic mice leads to increased hair formation. Right panels depict Masson’s trichrome staining of skin sections (blue = dermal collagens, red = muscle and keratin). Scale bars = 200 µm. **b** Quantification of the number of anagen HFs per 2 mm skin section at P50. *n* = 5, male control +*/*+; *n* = 5, male Tg/Tg(Ctsl-GDNF), *p* ≤ 0.001; error bars = SEM, two-way ANOVA with Bonferroni post-hoc test. **c** Immunofluorescence analysis of GDNF expression in skin. Hair follicle (HF). See also Figs. S1–2. **d** GDNF promotes hair formation after depilation. Dorsal skins were wax depilated (outlined in white) and analyzed 4 days later. Top views of the depilated skins (upper panels), and H&E-stained tissue sections (lower panels). Hair shaft (arrow). Scale bars = 200 µm. **e** GDNF promotes the proliferative expansion of K15^+^ BSCs within HFs. Immunofluorescence analysis depicting representative HFs (outlined in white) at start (non-depilated; upper panels) and 1-day post depilation (lower panels). Magnifications of Ki67^+^ proliferating K15^+^ BSCs (bracket). Hair follicles (HF), hair germ (Hg). Scale bars = 20 µm. **f** qPCR analysis of GDNF-mediated hair formation 1 day after depilation. Two-tailed Student’s *t*-test (*p* ≤ 0.05*≤0.001**≤0.001***), *n* = 3 skin samples per group; error bars = SEM. **g** BSCs purified from hair follicles using FACS. CD34^+^/CD49f^+^ purification of BSCs (top). Quantification of BSC colonies treated with either vehicle (PBS) or GDNF (100 ng/ml) at 3 or 6 days after plating (middle). BSC colonies (bottom, arrows). Error bars = SEM, two-way ANOVA, *n* = 3, *p* < 0.001. Scale bar = 0.1 mm.

In order to study the function of GDNF in promoting self-renewal of spermatogonial stem cells, we had generated transgenic mice that overexpressed *Gdnf* driven by the lysosomal proteinase cathepsin L (*Ctsl*) gene promoter (herein referred to as Tg(Ctsl-Gdnf)) within somatic Sertoli cells^20^. Incidentally, one of the unexpected phenotypes was a profound effect on hair follicle formation in both male and female homozygous Tg(Ctsl-Gdnf) mice (Fig. 1a, b). At 50-days of age, HFs in wild-type mice are kept in the telogen resting stage of the hair cycle characterized by a miniaturization in structure. In contrast, homozygous Tg(Ctsl-Gdnf) mice exhibited anagen HFs characterized by increased growth of the shaft diameter and entry into the hypodermis. GDNF was found to be present predominantly within the interfollicular space and in the vicinity of sebaceous glands of HFs in wild-type mice (Supplementary Fig. 1). In comparison, homozygous Tg(Ctsl-Gdnf) mice exhibited increased GDNF concentrated at the base of sebaceous glands proximate the hair BSC compartment, and sequestered in the interfollicular space (Fig. 1c and Supplementary Fig. 1). Importantly, the expression pattern of GDNF in the HFs of Tg(Ctsl-Gdnf) mice is consistent with the expression pattern of *Ctsl* present within the uppermost bulge layers harboring the stem cell reserve closest to sebaceous glands (Supplementary Fig. 2)^29^.

**Fig. 2:**
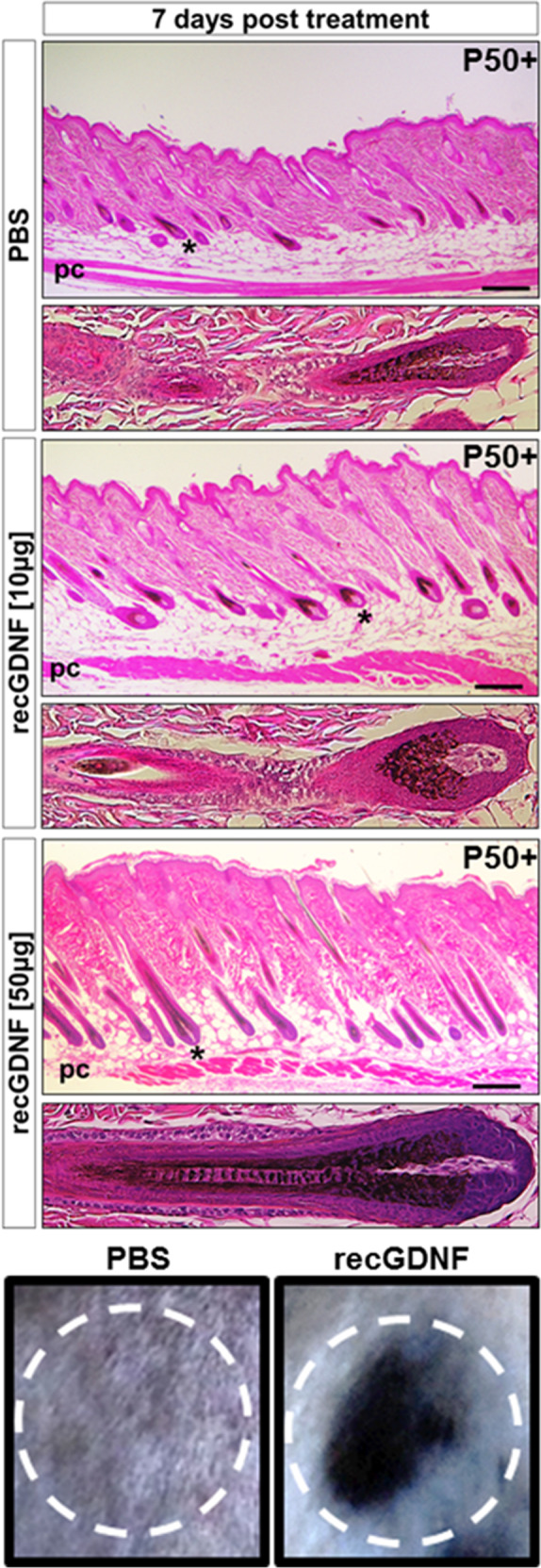
Recombinant GDNF initiates the anagen growth stage of the hair cycle. Dorsal skins of P50 C57BL/6J mice were injected with a single dose of 10–50 µg recGDNF or vehicle (PBS). Asterisk represents the HF in the expanded inset. Surface views of the vehicle and recGDNF-treated animals reveal prominent hair growth after rec-GDNF treatment only (lower panels). Panniculus carnosus (PC). Scale bars = 200 µm.

We investigated GDNF’s potential to regulate anagen induction by first monitoring responses to hair plucking (depilation) within the dorsal region of mice. At 4-days post depilation (dpd), control (+/+) animals exhibited early stage anagen HFs (Fig. 1d, left panels). However, homozygous Tg(Ctsl-Gdnf) mice resulted in elongated HFs with hair shafts and comparatively thicker skin (i.e., an indication of advanced anagen hair growth) by 4 dpd (Fig. 1d, right panels). Next, we appraised the effects of GDNF overexpression on BSCs during depilation-induced hair formation. Using immunofluorescence (IF), we observed no obvious differences in the expression of K15 in the non-depilated pool of BSCs between +/+ and homozygous Tg(Ctsl-Gdnf) HFs (Fig. 1e, top panels). At 1 dpd in control mice, we observed the expected proliferation (marked by Ki67) of hair germ cells (i.e., primed stem cells located between the bulge and Dp cluster) with limited proliferation of K15^+^ BSCs (Fig. 1e, left panels). In contrast, homozygous Tg(Ctsl-Gdnf) HFs exhibited a proliferative expansion and self-renewal of K15^+^ BSCs (Fig. 1e, right panels). The enhanced replenishment of the depleted BSC pool by elevated GDNF was validated using qPCR analysis, revealing a near-return of *K15* mRNA levels to non-depilated skin levels by 1 dpd (Fig. 1f). To further validate the self-renewing capacity of GDNF, we performed in vitro BSC colony formation assays using CD34^+^/CD49f^+^-purified BSCs derived from wild-type mice treated with or without recombinant GDNF (recGDNF). We observed a statistically significant (*p* < 0.01) increase in the size and growth rate of BSC colonies after treatment with recGDNF in comparison to vehicle treatment (Fig. 1g). Lastly, we ascertained if GDNF can activate anagen hair growth, per se, by monitoring hair formation starting from rest (telogen) upon administration of recGDNF to non-depilated 50-day-old C57BL/6J. We observed a dose-dependent response of recGDNF on HF formation and hallmarks of anagen (i.e., dermal thickening) compared to PBS-injected controls (Fig. 2), suggesting that GDNF can also activate hair BSCs in vivo to initiate anagen hair growth.

### GDNF-responding cells are present within the hair follicle bulge and dermal papillary compartments at distinct stages of the hair cycle

**Fig. 3:**
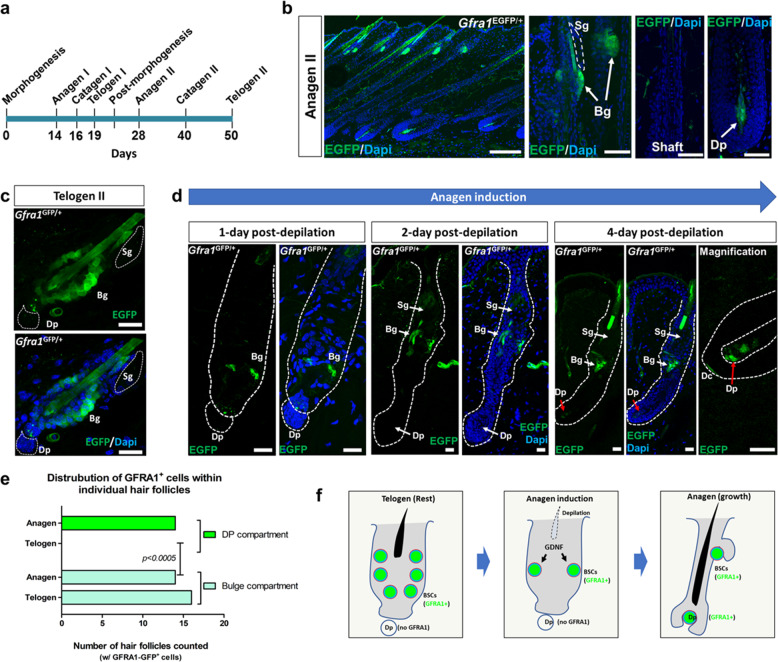
Temporal and spatial expression of GFRA1 dictates the function of GDNF-responding cells within hair follicles. **a** Schema depicting the morphogenic and post-morphogenic stages of the murine hair cycle. **b** GFRA1–EGFP is expressed within cells of the bulge (Bg) and dermal papillary (Dp) compartments of anagen HFs from *Gfra1*^*EGFP/+*^ mice. Scale bar = 200 µm (left panel). Scale bar = 50 µm (bulge, shaft, Dp panels). Sebaceous gland (Sg). See also Fig. S3. **c** GFRA1–EGFP is expressed within cells of the bulge (Bg) compartment but not within the dermal papillary (Dp) compartment of telogen HFs from *Gfra1*^*EGFP/+*^ mice. Bars = 20 µm. Sebaceous gland (Sg). **d** GDNF targets bulge (Bg) cells but not dermal papillary (Dp) cells of hair follicles during the early onset of hair growth. Time-course depilation analysis reveals that GDNF-responding cells are restricted to the bulge (Bg) compartment during the early stages of anagen induction (i.e., 1–2 days post depilation). However, by 4-days post depilation, GFRA1–EGFP becomes expressed within both Bg cells, and Dp cells that have become encased by matrix cells. At higher magnification, GDNF-responding cells also include dermal cap (Dc) cells at the base of Dp cells. Scale bars = 20 µm. Sebaceous gland (Sg). **e** Distribution of GFRA1–EGFP^+^ cells within hair follicles during telogen and anagen. Fisher’s exact test, two-sided, *p* = 0.0005. Dermal papilla (DP). **f** Model depicting GDNF targeting of BSCs during the early stages of hair formation.

To identify the GDNF-responding cells within the hair follicle, we utilized a *Gfra1* GFP reporter mouse line^23^. We generated heterozygous mice that expressed the *Gfra1 EGFP-*knock-in allele (*Gfra1*^*tm2.1Jmi*^) in a ubiquitous manner by crossing *Gfra1*^*tm2.1Jmi*^ mice to *β-actin* (ACTB)-cre mice. The resultant offspring herein are referred to as *Gfra1*^EGFP/+^. In this system, due to the ubiquitous expression of *ACTB*-cre, the EGFP reporter is controlled by the endogenous *Gfra1* promoter in every cell. We first monitored GFRA1–EGFP expression within anagen II HFs and observed EGFP^+^ cells specifically within the bulge and Dp compartments (Fig. 3a, b). In resting telogen II HFs, we observed EGFP^+^ cells within the outer bulge compartment, but not within the Dp compartment (Fig. 3c). These results were confirmed using immunofluorescence analysis with antibodies against K15, GFRA1, and RET within HFs of C57BL/6J mice (Supplementary Fig. 3).

To investigate GFRA1 expression during induction of anagen, we monitored GFRA1–EGFP expression soon after hair depilation using *Gfra1*^EGFP/+^ mice. At 1 dpd, we observed the expected reduction in the amount of EGFP^+^ cells localized to the bulge compartment (Fig. 3d, left 2 panels) compared to non-depilated HFs at rest (Fig. 3c). At the onset of anagen 1 dpd, we observed no EGFP^+^ cells localized to the Dp compartment (Fig. 3d, left 2 panels). At 2 dpd, a stage in which the Dp compartment first becomes encased by rapidly proliferating HF matrix cells^3^, we observed the expected downward growth of HFs into the dermis^3^, and the continued detection of EGFP^+^ cells within the bulge compartment alone. These results are consistent with our GDNF overexpression studies monitoring hair bulge stem cell expansion (Fig. 1e, g). At 4 dpd, the presence of EGFP^+^ cells in the bulge compartment persisted; however, at this time point we also detected GFRA1–EGFP expression within the Dp compartment of maturing anagen HFs (Fig. 3d, right panel). Lastly, we analyzed the distribution of GFRA1–EGFP^+^ cells within individual HFs during the telogen and anagen stages of the hair cycle. We observed a statistically significant difference (Fisher’s exact test) in the GFRA1–EGFP cellular distribution pattern within HFs in which GFRA1 is expressed in dermal papillary cells only during the anagen stage (Fig. 3e). However, GFRA1–EGFP+ cells are present within the HF bulge compartment in both telogen and anagen stages. Overall, these results are consistent with a model in which GDNF-responding cells within the bulge compartment and the DP are involved in the induction of anagen and growth of HFs, respectively (Fig. 3f).

### RET signaling within BSCs is required for the initiation of the hair cycle and depilation-induced hair regeneration

**Fig. 4:**
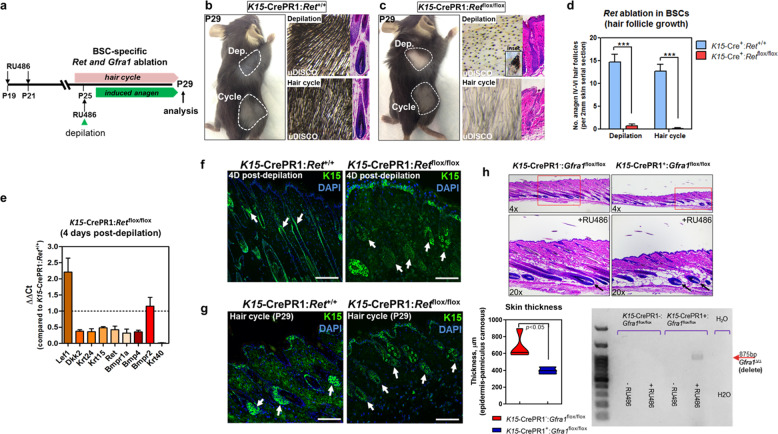
RET signaling in BSCs is required for anagen induction and depilation-induced hair formation. **a** Conditional deletion of *Ret* and *Gfra1* within BSCs. **b** Regrowth of HFs in control mice after depilation and demonstration of presence of anagen HFs at P29. Outlined in white are the uDISCO-cleared regions used for analysis. The hair cycle region was shaved immediately before imaging and tissue collection. H&E staining of the regions are shown in the insets. Dep (depilated). **c** Severe hair phenotypes in BSC-specific *Ret*-deleted mice after depilation and at P29. uDISCO-cleared depilated skin samples lack mature HFs that retains pigmentation (inset) (upper right panel). uDISCO-cleared skin samples at P29 depict failure of HFs to transition into the anagen stage (lower right panel). H&E staining of the regions are shown in the insets. **d** Quantification of the number of anagen HFs present within histological skin sections at 4-days post depilation and at P29. *n* = 4, male control *K15*-CrePR1^+^:*Ret*^+/+^; *n* = 4, male mutant *K15*-CrePR1^+^:*Ret*^flox/flox^; ***p ≤ 0.001; error bars = SEM, two-way ANOVA with Bonferroni post-hoc test. **e** qPCR analysis at 4-days post depilation. Data represents *K15*-CrePR1^+^:*Ret*^flox/flox^ *versus K15*-CrePR1^+^:*Ret*^+/+^ depilated skin samples. *n* = 4–5 male skin samples per group. **f** K15 expression in control and BSC-specific *Ret*-deficient skin at 4-days post depilation. K15 expression within the BSC population (arrows) in control (left panel) and *Ret*-deficient (right panel) hair follicles shows loss of K15^+^ cells after *Ret* ablation. Scale bars = 200 µm. **g** K15 expression in control and BSC-specific *Ret*-deficient skin during anagen at P29. BSC population (arrows) in control (left panel) and *Ret*-deficient (right panel) hair follicles shows loss of K15^+^ cells after *Ret* ablation. Scale bars = 200 µm. **h** Conditional deletion of *Gfra1* within BSCs. H&E-stained skin sections at 9 days post *Gfra1* ablation(top panel). Arrows depict delayed encapsulation of dermal papillae upon *Gfra1* ablation within BSCs. Quantification of the skin thickness from the epidermis to the underlying panniculus carnosus muscle (lower panel). *n* = 4, male control *K15*-CrePR1^-^:*Gfra1*^flox/flox^; *n* = 3, male conditional knockout *K15*-CrePR1^+^:*Gfra1*^flox/flox^; *p* ≤ 0.05; error bars = SEM, Student *t*-test. PCR confirmation of deletion of the Gfra1 allele after RU486 treatment (lower right panel).

To study the role of downstream signaling by GDNF during the hair cycle and depilation, we conditionally deleted the signal transducing receptor, *Ret*, specifically within BSCs by generating *K15*-CrePR1:*Ret*^flox/flox^ mice (Fig. 4a). In this genetic model system, upon progesterone antagonist mifepristone exposure (topical 1% synthetic RU486), Cre recombinase is expressed in K15^+^ BSCs to initiate Cre-mediated excision of the floxed exons 14 and 15 of the RET kinase domain to generate non-functional RET^30^. As expected, in control *K15*-CrePR1:*Ret*^+/+^ mice we observed underlying pigmented skin and HFs at the second anagen stage (P29) of the hair cycle through histological means that also incorporated an ultimate 3D imaging of solvent-cleared organs (uDISCO) whole-mount clearing method (Fig. 4b, lower right panel)^31^. At 4 dpd (i.e., starting at P25), in control animals we observed the expected regrowth of anagen HFs (Fig. 4b, upper right panel). However, when *Ret* was conditionally deleted in BSCs during telogen (P19-21), the HFs remained at rest and failed to launch into the anagen hair cycle by P29 (Fig. 4c, lower right panel). Furthermore, *Ret* ablation within BSCs prevented the reconstitution of the lower HF after depilation (Fig. 4c, upper right panel). Histomorphometric analysis revealed a statistically significant decrease in the number of anagen HFs present both after depilation and at P29 within *K15*-Cre:*Ret*^flox/flox^ animals versus controls (Fig. 4d). qPCR analysis comparing 4 dpd skin samples derived from *K15*-CrePR1:*Ret*^flox/flox^ mice versus control samples in anagen confirmed the reduction in *Ret* and *K15* transcripts after *Ret* deletion, and absence of mature HFs and differentiated cuticular keratinocytes through reduced *Krt40* (keratin 40) mRNA levels (Fig. 4e). The qPCR analysis also revealed enhanced WNT (i.e., as shown by increased *Lef1* and decreased *Dkk2* expression) and reduced BMP (i.e., as evidenced by reduced *Bmp4*/*Bmpr1a*) signaling within RET-deficient skin (Fig. 4e), suggesting a comparable inhibition in the hair cycle. Interestingly, despite the BSC-specific RET-mediated defect in hair formation after depilation, individual HFs did retain melanin production (Fig. 4c, upper right panel, inset), presumably due to intact RET tyrosine kinase signaling within melanocytes^26^. As expected in *K15*-CrePR1:*Ret*^+/+^ control skin, immunofluorescence analysis revealed K15 expression predominantly within expanding BSCs along the proximal portion of actively growing HFs in depilated samples (Fig. 4f, left panel). However, BSC-specific *Ret* ablation resulted in punctate expression of K15 in addition to areas void of any K15 expression among individual hair follicles after depilation (Fig. 4f, right panel). Certain hair follicles also entirely lacked K15-expressing BSCs after depilation, suggesting a range of BSC effects upon *Ret* ablation (Fig. 4f, right panel). During the anagen stage of the hair cycle (P29), we observed the expected clusters of K15^+^ BSCs within control HFs (Fig. 4g, left panel). However, in *K15*-CrePR1:*Ret*^flox/flox^ HFs, we observed reduced numbers of K15^+^ cells within individual HFs at the expected anagen stage of the hair cycle (Fig. 4g, right panel). These results suggest that RET signaling within BSCs is required to germinate the lower HF to the next anagen stage.

In order to study the role of GFRA1, one of the RET co-receptors of GDNF, specifically within BSCs, we generated *K15*-CrePR1:*Gfra1*^flox/flox^ mice^32^ (Fig. 4h). To ablate *Gfra1*, we topically applied 1% RU486 and then monitored hair growth at the expected anagen stage. Compared to control (*Gfra1*^flox/flox^), *Gfra1* ablation within BSCs resulted in a suppression, but not prevention of hair growth as reflected by a statistically significant decrease in skin thickness and presence of shorter HFs (Fig. 4h). Furthermore, in comparison to control HFs, dermal papillae were not entirely encapsulated upon *Gfra1* ablation, suggesting a delay in the hair cycle^3^ (Fig. 4h, arrows). These results suggest that alternative co-receptors in combination with GFRa1 are likely involved in initiating the hair cycle mediated by downstream RET signaling.

### RET signaling within interfollicular epidermal keratinocytes and outer epithelial cells of hair follicles is not required for hair cycle progression and plucking-induced anagen development

**Fig. 5:**
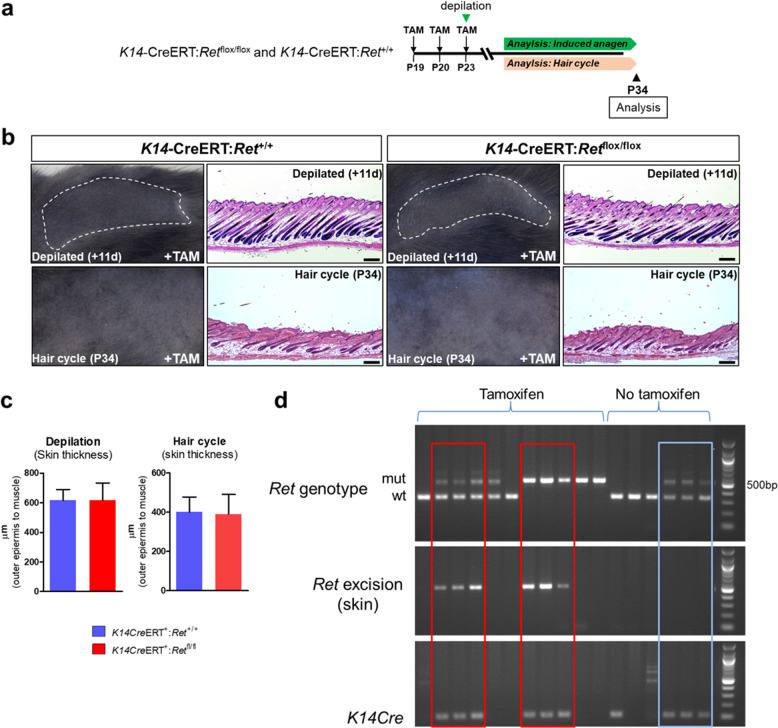
*Ret* deletion within K14-expressing epithelial cells does not affect onset of anagen or induced anagen development after depilation. **a** Overall experimental design to study RET signaling within K14-expressing cells during the start of the hair cycle and following plucking-induced anagen development. **b** Macroscopic and microscopic H&E analysis of hair follicles after *Ret* ablation in K14+ cells. In the upper left panels, depilated regions of the animals are marked by dashed lines. In the lower left panels, unperturbed skins were used to assess the effect of *Ret* ablation on the hair cycle, and only shaved during imaging and tissue collection at P34. H&E sections are in the right panels. Bars = 200 μm. **c** Quantitative analysis of *Ret* ablation within K14+ cells. Skin thickness was accessed by measuring the distance from the epidermis to the underlying panniculus carnosus muscle. *n* = 3 male *K14*-CreERT^+^:*Ret*^+/+^ and *K14*-CreERT^+^:*Ret*^flox/flox^ mice per group. error bars = SEM, Student *t*-test. **d** Cre-mediated excision of exons 14–15 of *Ret* after tamoxifen treatment. PCR was used to determine *Ret* deletion in the skin. *Ret* deletion was observed only in tamoxifen-treated *K14*-CreERT^+^:*Ret*^flox/flox^ and *K14*CreERT^+^:*Ret*^flox/+^ mice (red boxes).

In order to further characterize the cellular specificity of RET signaling during HF cycling and induced anagen development, we conditionally ablated *Ret* within both interfollicular basal epidermal keratinocytes and cells that make up the outer epithelial layer of HFs^33^. These cells express keratin 14 (K14) and play important roles in maintaining the lipid barrier as well as anagen development of HFs^34^. To ablate *Ret* within basal epidermal keratinocytes and outer epithelial cells of HFs, we generated K14CreERT:*Ret*^flox/flox^ mice and induced CRE-mediated recombination by administration of tamoxifen (Fig. 5a). At 11-days after synchronized anagen induction by wax depilation, we observed no difference in hair shaft formation as a macroscopic indicator of advanced anagen development between control and experimental K14-*Ret*-mutant animals (Fig. 5b, upper left panels). Similarly, histological analysis revealed that anagen progression was no different between control and experimental K14-*Ret*-mutant animals after depilation (Fig. 5b, upper right panels). Changes in the natural hair cycle were also investigated after *Ret* ablation within K14-postivie cells. Once again, we observed no difference in natural anagen development of HFs following tamoxifen treatment between control or K14-*Ret-*mutant mice both at the macroscopic and microscopic levels (Fig. 5b, lower panels). These observations were confirmed by quantifying the thickness of the skin, which is an indicator of the degree of anagen progression^3^ (Fig. 5c). Lastly, Cre-mediated deletion of the floxed *Ret* allele was confirmed via deletion PCR using harvested skin tissues (Fig. 5d). Overall, our data show that RET signaling within interfollicular basal epidermal keratinocytes and outer epithelial cells of HFs is not required for hair cycle progression and induced anagen development in postnatal mice.

### GDNF accelerates skin wound repair and healing with enhanced hair formation

Given the interrelationship between hair follicles and cutaneous wound repair and healing^9^, we investigated the effects of GDNF toward repair and healing of 3 mm full-thickness truncal wounds in mice.

**Fig. 6:**
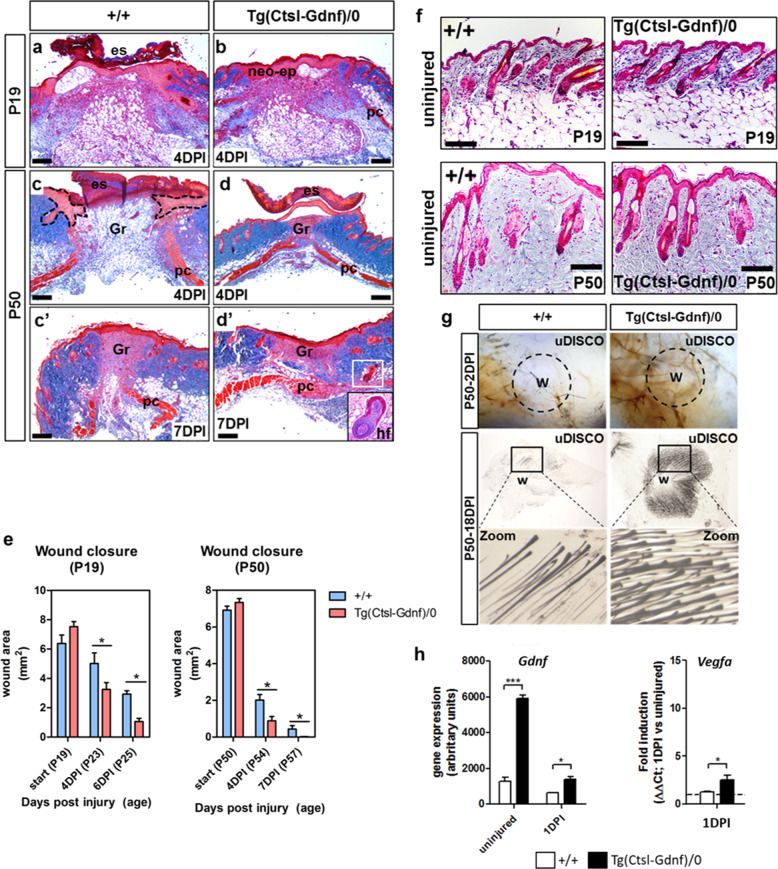
GDNF overexpression promotes cutaneous wound repair and healing associated with hair formation. **a** Wound repair in P19 non-transgenic (+/+) mice at 4 DPI. Staining was done with Masson’s trichrome stain (Blue = collagen types, red = keratin and muscle). Scale bar = 200 µm. **b** Wound repair in P19 hemizygous Tg(Ctsl-Gdnf)/0 mice at 4 DPI. Masson’s trichrome. Scale bar = 200 µm. **c**, **c’** Wound repair and healing in P50 non-transgenic (+/+) mice at 4–7 DPI. Masson’s trichrome. Scale bar = 200 µm. **d**, **d’** Wound repair and healing in P50 hemizygous Tg(Ctsl-Gdnf)/0 mice at 4–7 DPI. Masson’s trichrome. Scale bar = 200 µm. **e** Quantification of wound closure in non-transgenic and hemizygous Tg(Ctsl-Gdnf)/0 mice. Wound size measured at 0–7 DPI: P19 set, non-transgenic, *n* = 4; hemizygous Tg(Ctsl-Gdnf)/0, *n* = 7. P50 set, non-transgenic, *n* = 6; hemizygous Tg(Ctsl-Gdnf)/0, *n* = 8. *p ≤ 0.05; error bars = SD, two-way ANOVA with Bonferroni post-hoc test. **f** Hair follicle stage within uninjured skin. Masson’s trichrome. Scale bar = 100µm. **g** Peri-wound hair formation assessed using the uDISCO clearing method in P50 mice. Wound (W) blood vessel formation depicted at 2 DPI (upper panel). Peri-wound hair formation assed at 18 DPI (lower panels). **h** Enhanced *Gdnf* and *Vegfa* expression in injured Tg(Ctsl-Gdnf)/0 wounds. Real-time qPCR was performed at 1 DPI using non-transgenic and Tg(Ctsl-Gdnf)/0 wounds. Non-transgenic and hemizygous Tg(Ctsl-Gdnf)/0, *n* = 5. *p ≤ 0.05, ***p ≤ 0.001; error bars = SD, Student’s *t*-test.

**Fig. 7:**
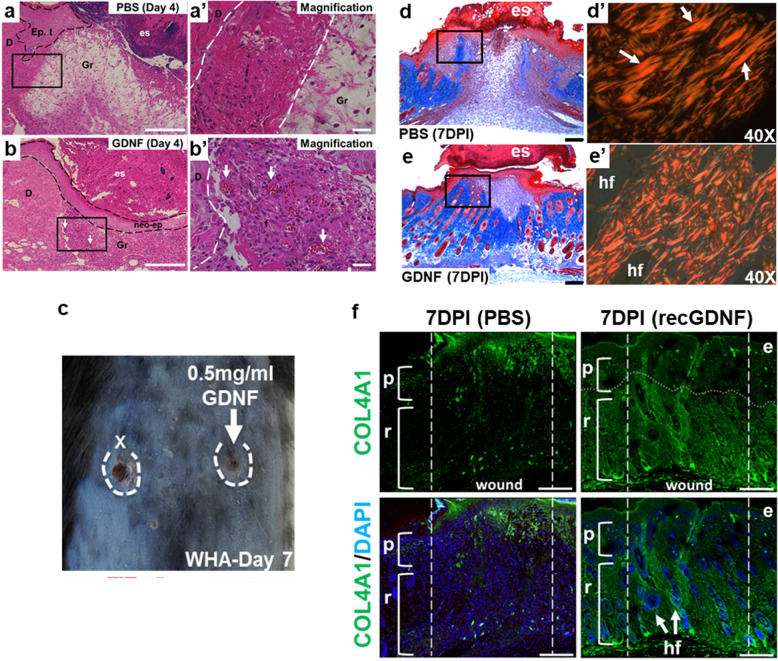
Recombinant GDNF promotes skin repair and healing after injury. **a**, **b** recGDNF accelerates wound re-epithelization and granulation tissue maturation by 4 DPI. Vehicle-treated wounds depicted by H&E staining (panels **a**, **a”**). Epithelial tongue (Ep. t), granulation tissue (Gr), eschar (es). recGDNF-treated wounds (50 µg/wound) formed the neoepidermis (Neo-ep) by 4 DPI (**b**). Appearance of vascularized red blood cells (arrows) within granulation tissue (panel **b’**). Dermis (D). Scale bars = 200 µm (**a**, **b**), and 100 µm (**a’**–**b’**). **c** Recombinant GDNF improves dorsal skin wound closure by 7 DPI. Single intrawound injection of 33 µM recGDNF (50 µg recGDNF in 100 µl PBS). Vehicle (x injected site). **d**, **e** Improved skin healing after recGDNF treatment by 7 DPI. Masson’s trichrome stain (Blue = collagen types, red = keratin and muscle) of vehicle-treated wound sections (panel **d**). Polarized light microscopy of picrosirius red stained control wound sections reveal large parallel bundles of collagen (arrows) analogous to scar formation (**d’**). In recGDNF-treated wounds, reduction and maturation of the granulation tissue with hair follicle (hf) formation (**e**), coupled with resolution of scar tissue into interwoven collagen bundles (**e’**). Scale bars = 200 µm (**d**, **e**). See also Fig. S4. **f** Restoration of COL4A1 expression in the reticular dermis at the wound site after recGDNF treatment. COL4A1 immunofluorescence staining at 7 DPI (upper panels). The dotted vertical lines encompass the wound region. The papillary (p) dermis is the uppermost layer of the dermis, while the reticular (r) layer is the lower layer of densely packed collagen fibers. The horizontal dotted line in the recGDNF-treated sample separates the papillary and reticular dermal layers based on COL4A1 expression. DAPI and COL4A1 merged images (lower panels). Epidermis (e), hair follicle (hf). COL4A1 expression of uninjured skin is depicted in Driskell et al. 2013^37^. Scale bars = 200 μm.

To control for increased cellularity due to GDNF overexpression in the homozygous state at the start of the experiments (Fig. 1a), we performed wound healing assays using hemizygous Tg(Ctsl-Gdnf)/0 mice. Hemizygous Tg(Ctsl-Gdnf)/0 P19 and P50 mice show accelerated wound re-epithelization, eschar detachment, neoepidermal formation and enhanced panniculus carnosus (i.e., the deep striated muscle) reformation compared to their non-transgenic counterparts by 4–7 days post injury (DPI) (Fig. 6a–e). Interestingly, we observed ectopic anagen HFs within the hypodermis of 50-day-old Tg(Ctsl-Gdnf)/0 mice after injury when HFs are expected to be at rest (Fig. 6d’, inset). Importantly, hemizygous Tg(Ctsl-Gdnf)/0 mice exhibit no baseline difference in the hair follicle stage (i.e., telogen) when compared to control at both ages (Fig. 6f). Using uDISCO, we observed increased blood vessel networks within wounded hemizygous Tg(Ctsl-Gdnf)/0 mice (Fig. 6g, upper right panel). At 18 DPI, we observed enhanced HF formation at peri-wound regions upon GDNF overexpression in telogenic mice (Fig. 6g, lower panels), suggesting that GDNF has the potential to influence either uninjured pre-existing or injured/amputated HFs in their recovery. Gene expression analysis revealed a statistically significant increase in *Vegfa* transcript in Tg(Ctsl-Gdnf)/0 mice when compared to non-transgenic animals at 1 DPI (Fig. 6h), suggesting early onset of vascular endothelial cell proliferation mediated by GDNF overexpression. We next appraised wound repair and healing in P50 C57BL/6J mice treated with vehicle (PBS) or recGDNF (50 µg per intrawound injection) up to 11 DPI. By 4 DPI, treatment with recGDNF resulted in accelerated re-epithelization of wounds with concomitant maturation of the granulation tissue to fill the injured area (Fig. 7). There were increased deposits of vascularized red blood cells within the recGDNF-treated wound bed when compared to control (Fig. 7a’, b’). By 7 DPI, the ability of recGDNF to accelerate wound closure was visually apparent (Fig. 7c). Histologically, the PBS-treated wounds had yet to fully re-epithelialize but contained loosely packed granulation tissue in the wound bed (Fig. 7d). In contrast, recGDNF-treated wounds were fully re-epithelialized with neoepidermal detachment from the overlying eschar (Fig. 7e). In addition, there was shrinkage of the granulation tissue (i.e., assessed by Trichrome staining) and appearance of HFs by 7 DPI. We investigated the effect of recGDNF on collagen fiber organization and architecture using picrosirius red staining coupled with polarized light microscopy to compare mixed birefringence. We compared multiple collagen-enriched regions adjacent to the wound bed (Supplementary Fig. 4), and in PBS-treated wounds we observed large red-orange birefringent type 1-containing collagen fibers arranged in parallel scar-like orientation (Fig. 7d’)^35^. In contrast, after recGDNF-treatment we observed smaller fibers with a mixture of red-orange and green birefringence (i.e., indicative of loosely packed type III collagen^35^) (Fig. 7e’). Furthermore, the fibers were occasionally arranged perpendicular to one another analogous to uninjured skin (Supplementary Fig. 4c)^36^. Importantly, the lower reticular half of the dermal compartment of skin exhibited re-emergence of type IV collagen after recGDNF-treatment analogous to uninjured skin compared to PBS-treated wounds (Fig. 7f)^37^. Collectively, injured skin healed as a scar in controls, while recGDNF-treatment promoted tissue resolution comparable to unwounded skin.

### GDNF enhances BSC contribution to cutaneous wound closure

**Fig. 8:**
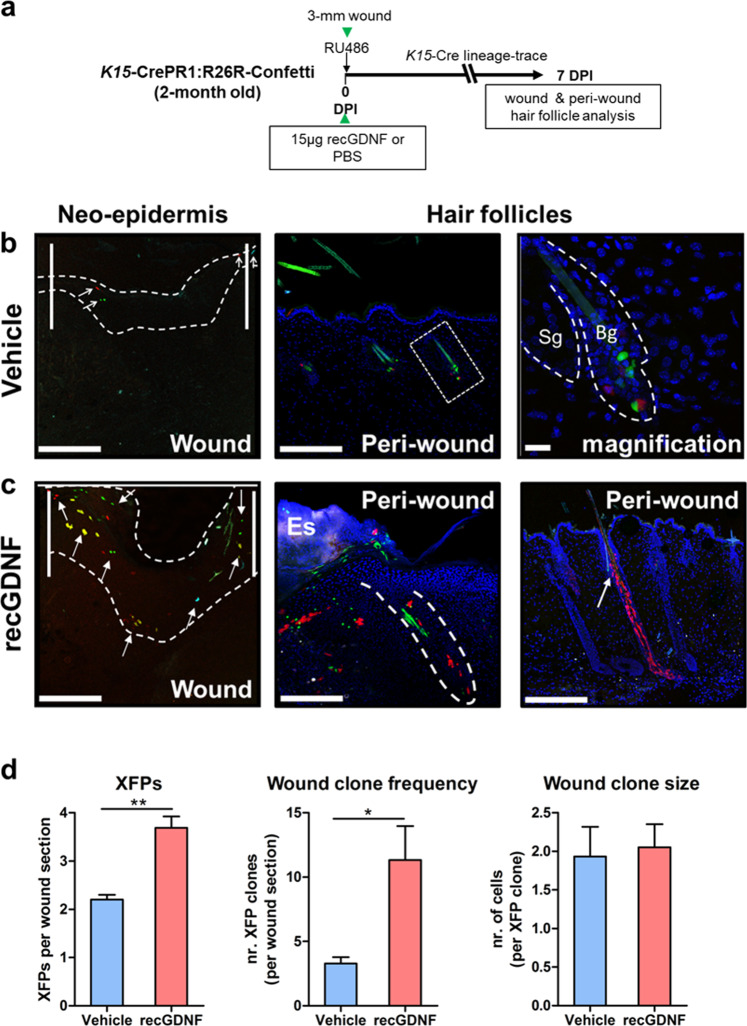
GDNF signaling improves cutaneous wound repair through expansion and recruitment of hair bulge stem cell epidermal progenitors in mice. **a** Experimental design using the *K15*-CrePR1:R26R-Confetti multi-label reporter line to study the effect of recGDNF on the fate of cells derived from BSCs after injury. **b** Commitment of BSC-derived keratinocytes to the injured neoepidermis in vehicle-treated mice. Neo-epidermis (dotted line) at the wound site marked off by the white vertical bars (left panel). Confetti-labeled keratinocytes (arrows) are present in the neoepidermis by 7 DPI. Quiescent peri-wound HFs (boxed) that show initial Confetti labeling of BSCs (magnification) by 7 DPI (middle and right panels). Bulge (Bg), sebaceous glands (Sg). Scale bars = 200 μm; scale bar (magnification) = 20 μm. **c** Recombinant GDNF promotes the recruitment of BSC progeny to the injured neoepidermis by 7 DPI. Increased migration and commitment of GDNF-stimulated BSC-derived progeny to the wound neoepidermis (left panel). Cohesive streaks of BSC-descendants recruited to the wound site (middle panel). GDNF-mediated activation of BSCs (arrow) that give rise to RFP^+^ cells which define a HF (right panel). Es (eschar). Scale bars = 200 μm. **d** Quantification of Confetti-labeled cells in the neoepidermis after injury. Left graph depicts the individual fluorescent proteins (termed XFPs) observed within the neoepidermis by 7 DPI. Middle graph depicts the number of individual clones (i.e. represented by cohesive single XFPs) present in the injured neoepidermis. The right graph depicts the number of cells within a clone at the injured neoepidermis. For statistics, a total of four serial sections through the wound midline were averaged and assessed per mouse at 7 DPI. Vehicle-treated, *n* = 3 male mice; recGDNF-treated, *n* = 4 male mice. *p = 0.04, **p = 0.003; error bars = SEM, two-tailed Student’s *t*-test. Days post depilation (DPI).

We hypothesized that GDNF signaling underlies BSC activation and commitment of their progenitors to the neoepidermis during wound repair. To assess the contribution of BSCs and their progenitors after skin injury, we applied a multicolor lineage tracing strategy using the *K15*-CrePR1:R26R-Confetti mouse model. After *K15*-specific Cre-mediated recombination, each cell descendant will stochastically express one of four fluorescent proteins to generate nuclear GFP, cytoplasmic YFP or RFP, or membranous CFP-labeled cells^38^. This system allows for the discrimination between the clonal progeny of individual cells derived from BSCs after recGDNF or vehicle treatment over time. After a single topical application of 1% RU486 to induce Confetti labeling, we wounded 2-month-of-age mice and traced the BSC progeny for 7 days (see Fig. 8a for an overview). In vehicle-treated animals, relatively few Confetti-labeled cells were observed closer to the wound margins and were restricted to the outer non-basal layer of the neoepidermis within the experimental window (Fig. 8b, left panel). In corroboration, we observed resting follicles with Confetti-positive cells restricted to BSC compartments, signifying relative inactivity after vehicle treatment (Fig. 8b, middle and right panels). In contrast, recombinant GDNF promoted the activation and transient commitment of BSC-derived Confetti-labeled cells to a variety of structures within the neoepidermis, including the outer differentiated and lower basal layers, by 7 DPI (Fig. 8c left panel). We frequently detected Confetti-labeled peri-wound HFs when treated with recGDNF, suggesting that GDNF can activate resident BSCs, whereby the daughter cells give rise to entire follicles (Fig. 8c, middle and right panels). Within serial wound sections, we quantified the number of uniquely colored fluorescent labels (termed XFPs with a maximum of four), the number of cohesively associated cells in the same color as clones, and the number of cells among a single clone within neoepidermal tissues (Fig. 8d). In vehicle-treated wound samples, we observed on average 2.2 (±0.1 SEM) XFPs, 3.2 (±0.4 SEM) clones, and 2.0 (±0.2 SEM) cells within individual clones after injury per wound neoepidermal section. In contrast, recGDNF treatment yielded on average a statistically significant increase of 3.7 (±0.2 SEM; *p* = 0.003 versus. vehicle) XFPs and 11.3 (±2.6 SEM*; p* = 0.04 versus. vehicle) clones, and 1.9 (±0.3 SEM; not significant) cells within clones of the neoepidermal region after injury per section.

### BSC-specific RET deficiency leads to impaired cutaneous wound closure and healing

**Fig. 9:**
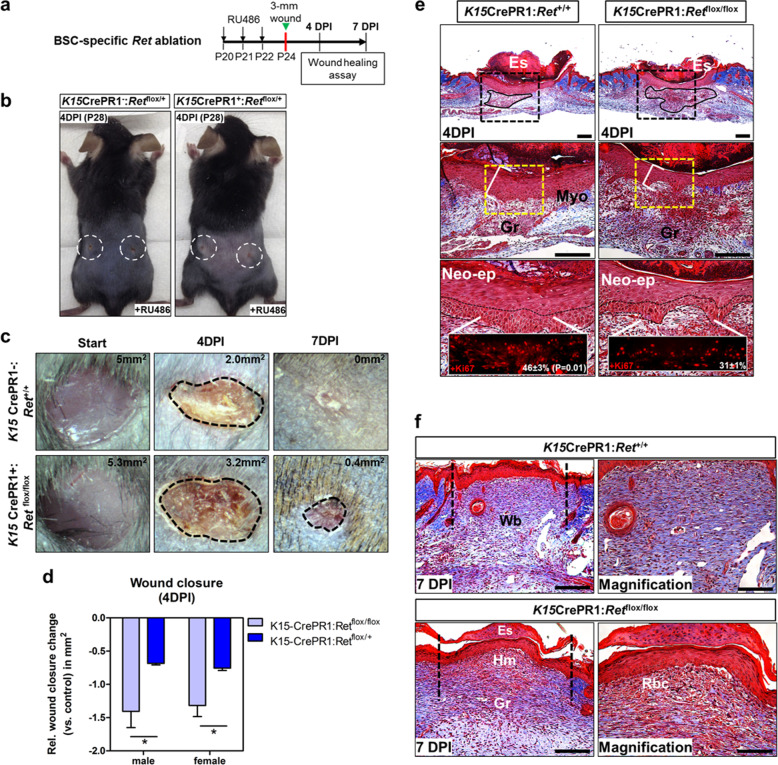
*Ret* ablation within BSCs impairs wound repair and healing in mice. **a** Schema depicting the experimental design to investigate the role of RET signaling in BSCs during cutaneous wound healing. Two 3 mm full-thickness skin punctures were administered in the lower truncal region for each mouse. **b** Defective hair formation in *K15*CrePR1:*Ret*^flox/+^ mice. Prior to studying wound healing responses (wounds encircles in white) by 4 DPI, heterozygous *Ret*-deficient mice exhibited defects in underlying skin pigmentation after 1% RU486 treatment^3^. **c** Impaired wound closure upon BSC-specific ablation of RET signaling. Top views of wounds in RU486-induced *Ret*-ablated mice at 4–7 DPI with results of surface area calculations. **d** Quantification of cutaneous wound closure upon BSC-specific *Ret* deletion relative to control at 4 DPI. For statistics, we averaged the surface areas for two wounds per mouse by 4 DPI. Males: controls, *n* = 3; *K15*-CrePR1:*Ret*^flox/flox^, *n* = 3; *K15*-CrePR1:*Ret*^flox/+^, *n* = 3. Females: controls, *n* = 3; *K15*-CrePR1:*Ret*^flox/flox^, *n* = 5; *K15*-CrePR1:*Ret*^flox/+^, *n* = 3. *p = 0.04; error bars = SEM, two-tailed Student’s *t*-test. **e** Neoepidermal hypoplasia and delayed granulation tissue maturation in injured *K15*-CrePR1:*Ret*^flox/flox^ mice by 4 DPI. Compared to control mice (left panels), *Ret* ablation was associated with impaired granulation tissue maturation (outlined in black; upper right panel, boxed region). Increased thickness of the neoepidermal layer (boxed in yellow) in control wounds compared to *Ret*-deficient wounds by 4 DPI (middle panels). Masson’s trichrome stain (Blue = collagen types, red = keratin and muscle). Percentage of Ki67^+^ proliferative keratinocytes within the basal layer of the neoepidermis (lower panels). *K15*CrePR1:*Ret*^+/+^, *n* = 3 serial sections; *K15*CrePR1:*Ret*^flox/flox^, *n* = 3 serial sections. *p = 0.01; error bars = SD, two-tailed Student’s *t*-test. Bars = 200 μm. Eschar (Es), granulation tissue (Gr), neoepidermis (neo-ep), myofibroblast (myo), days post injury (DPI). **f** Skin wound healing defects in *K15*-CrePR1:*Ret*^flox/flox^ mice at 7 DPI. Control wounds have detached eschar from the neoepidermis (upper panel); however, BSC-specific *Ret*-ablated mice fail to detach the eschar (lower panel) (Scale bars = 200 μm). Right panels depict magnification of the wound bed (Wb) regions, whereby *Ret*-mutant wounds exhibit increased red blood cell (Rbc) exudate from damaged capillaries (Scale bars = 100 μm). Masson’s trichrome stain. Granulation tissue (Gr), hematoma (Hm).

Because GDNF-mediated signaling within BSCs was found to be involved in epidermal repair after injury, we hypothesized that ablation of GDNF signaling within BSCs may inhibit the repair and healing processes. To test this, we performed skin puncture assays utilizing the *K15*-CrePR1:*Ret*^flox/flox^ mouse line. We induced Cre-mediated *Ret* deletion in 20-day-old mice by topical pretreatment with 1% RU486 followed by creation of 3 mm full-thickness truncal wounds (Fig. 9a). We observed a reduction in the underlying hair in heterozygous *K15*-CrePR1:*Ret*^flox/+^ mice at 4 DPI (P28) (Fig. 9b). In control *K15*-CrePR1:*Ret*^+/+^ animals, we macroscopically observed complete wound closure by 7 DPI; however, *K15*-CrePR1:*Ret*^flox/flox^ mice remained incompletely healed by this time point (Fig. 9c). We observed a statistically significant gene dosage effect on wound size after *Ret* deletion within BSCs (Fig. 9d). Relative to controls, we observed a −1.4 ± 0.2 mm^2^ and −0.6 ± 0.02 mm^2^ reduction (*p* = 0.04) in the wound size at 4 DPI within male homozygous and heterozygous *Ret*-mutant mice, respectively. Female *Ret*-mutant mice also showed a similar pattern of decreased wound closure relative to control mice (i.e., −1.3 ± 0.16 mm^2^ [homozygous] and −0.7 ± 0.02 mm^2^ [heterozygous] *p* = 0.04, respectively). The decrease in wound closure rates in *K15*-CrePR1:*Ret*^flox/+^ and *Ret*^flox/flox^ mice was associated with impaired granulation tissue maturation when compared to control provisional wound matrix at 4 DPI (Fig. 9e). In control wounds, there was uniform infiltration of the granulation tissue assessed by Trichrome staining (Fig. 9e, middle left panel) that was less organized in the mutants (Fig. 9e, middle right panel). Compared to controls, we also observed neoepidermal hypoplasia in *K15*-CrePR1:*Ret*^flox/flox^ mice at 4 DPI due to an overall 15 ± 2% decrease (*p* = 0.01) in the proliferation of basal keratinocytes (Fig. 9e, bottom panels). Delayed wound healing persisted up to 7 DPI due to lack of RET in BSCs, resulting in failure of the eschar to entirely detach from the neoepidermis, and impaired granulation tissue resolution with presence of superficial hematoma containing non-vascularized blood cells (Fig. 9f, bottom panel). Overall, RET signaling promotes the contribution of reparative cells to the neoepidermis derived from BSCs, whereby genetic ablation of *Ret* in BSCs resulted in a concomitant delay in wound repair and healing processes in mice.

## Discussion

Our findings show that GDNF promotes hair formation and cutaneous would healing in mice. Both recombinant and transgene overexpression of GDNF promoted BSC colony growth, hair formation, and skin repair after wounding, while genetic ablation of *Ret within* BSCs prevented the onset of the hair cycle, the anagen induction of depilated hair follicles, and reduced the rate of wound closure. GDNF has previously been suggested to play a role in the natural hair cycle^39^, however its function within BSCs has never been tested. Our data show that GDNF protein is present predominantly within the interfollicular space and in the vicinity of sebaceous glands, while single-cell sequencing results from mouse skin reveal that *Gdnf* transcripts are present in both bulge hair follicle stem cells, as well as keratinocytes of the interfollicular epidermis (Joost et al. 2016), suggesting that GDNF could be acting on more than one cell type within the skin. GDNF and its receptor GFRA1 are also expressed in human skin and show an age dependent decline^40^.

GDNF is known to form a receptor complex with GFRA1^41^ that signals through RET. We detected GFRA1 expression in BSCs using a genetic reporter as well as by immunofluorescence. *Gfra1* transcript was previously found to be enriched within K15^+^ BSCs^8,29^, and *Gfra1* transcripts increased during anagen, while reaching their lowest levels during the resting stage (telogen)^39^. Upon stimulation of the hair cycle, *Gfra1* transcript reappeared and increased over time in the natural setting. In the same study^39^, GDNF protein was reported to be increased after depilation, suggesting that GDNF might play a role in anagen induction within the natural setting. In addition to BSCs, we detected GFRA1-expression in Dp cells during the anagen growth phase following depilation. GDNF signaling within Dp cells may maintain HF growth, as global *Gfra1*^39^ and *Ret* knockout mice (Supplementary Fig. 5) exhibit premature catagen entry of HFs. However, it is still unclear if Dp cells are GDNF effector cells. Botchkareva et al. had postulated that GDNF-GFRA1 signaling within proximal and distal outer root sheath (ORS) and inner root sheath (IRS) cells of HFs may play a direct role in blocking premature catagen entry based on their *in situ* mRNA expression pattern of *Gfra1* and *Gdnf*^39^. Furthermore, *Gdnf* transcript was shown to peak during the catagen stage of the hair cycle, further supporting a role in promoting hair elongation and survival^39^. However, our studies found no evidence of GFRA1 protein within ORS and IRS cells, suggesting an alternative cellular mechanism which prevents hair regression by GDNF. Botchkareva et al. also reported that *Gfra1* mRNA was absent from Dp cells during all stages of the hair cycle. However, previous comparative transcriptomic studies revealed signatures in the Dp cell population of growing hair^42^, and these transcripts included *Gfra1* (i.e., 9.6-fold increase versus. matrix cells; 8.6-fold increase versus. ORS cells), which is consistent with our findings. Thus, this raises the possibility that GFRA1 signaling within other niche cells such as Dp cells may promote the maturation of HFs as recently shown for Dp sonic hedgehog signaling^43^, and whether or not GDNF signaling via Dp cells can affect wound healing as well.

Conditional ablation of *Ret* within BSCs prevented the onset of the hair cycle and anagen induction of depilated hair follicles, demonstrating the requirement of GDNF-mediated signaling through RET in BSCs. In contrast, while ablation of *Gfra1* among BSCs impaired the onset of the hair cycle, it had less of an effect than ablation of *Ret*, suggesting the possibility that other RET-coupled receptors may be involved in the hair cycle. Other members of the GDNF family (e.g., neurturin, persephin, and artemin) can also signal via RET but do so in combination with other co-receptors (GFRA2-4, respectively)^44^. Our *Gfra1*-reporter assay suggests the involvement of cells that are most likely affected by GDNF. In our *Ret* cKO studies, it is possible that the hair loss associated with *Ret* ablation may be, in part, also due to impaired *Gfra2-4* and neuturin, persephin, and artemin signaling as well. However, BSCs neither express *Gfra3* and *4* (Supplementary Fig. 6), nor ligands persephin and artemin^29^. On the contrary, BSCs do express *Gfra2*^29^, suggesting the possibility that the GFRA2-neuturin network may also be compromised upon *Ret* ablation in addition to impairment of the GFRA1-GDNF network. This model is consistent with the unique hair phenotypes depicted upon either ablation of *Gfra1* or *Ret* within BSCs.

Given the expression of GDNF within the interfollicular epithelium, we asked whether RET-mediated signaling within the IFE was important for HF cycling and induced anagen development. Conditional ablation of *Ret* within both interfollicular basal epidermal keratinocytes and cells that make up the outer epithelial layer of HFs^33^ using a tamoxifen-inducible keratin 14 Cre driver had no observable effect on hair shaft formation or anagen progression. These observations were confirmed by quantifying the thickness of the skin, which is an indicator of the degree of anagen progression^3^. Overall, our data show that RET signaling within interfollicular basal epidermal keratinocytes and outer epithelial cells of HFs is not required for hair cycle progression and induced anagen development in postnatal mice. Nonetheless, it is possible that the absence of a phenotype may have been due to incomplete ablation of *Ret* within the IFE, or that GDNF acts on K14-negative cells within the IFE.

We also found that GDNF promotes cutaneous wound healing and repair by targeting BSCs. By lineage tracing K15^+^ BSCs after wounding, we show that one of the modes for GDNF-mediated cutaneous wound repair is through activation and changes to cell identity. In contrast, ablation of RET signaling specifically within BSCs delayed wound closure after skin injury. This phenotype points to possible perturbations in BSC numbers, migration, and/or progenitor commitment to the reconstituted epidermis and subsequent dysregulation of resident neoepidermal and neighboring cells. Neoepidermal keratinocytes generate and secrete a number of potent growth factors to properly repair the denuded wound surface and underlying wound bed in intracrine and paracrine manners^45^. Future studies will entail the purification, and molecular and functional characterization of the GDNF-primed cells at the neoepidermis to examine their “reparative and regenerative” properties in more detail. In addition, from our *Ret* knockout animal studies, we provide evidence that GDNF signaling can also prevent HF regression, although the role of BSCs *versus* Dp cells in the process remains unclear. Notwithstanding wounding, damaged HFs endure premature regression to limit HF growth^46^, whereby active GDNF-RET signaling may also enhance survival of peri-wound HFs to improve overall wound outcomes.

In part, skin wound healing recapitulates morphogenesis, which entails the initiation of a developmental “reprogramming” cascade to promote cutaneous lineages that restores tissue integrity and function to its original state^14^. In injured adult tissue, scar formation ultimately results from excess accumulation of an unorganized ECM, whereby normal ECM architecture is rarely achieved during the healing process. In contrast to adult wounds, early gestation fetal skin wounds repair rapidly and heal with the absence of scar formation^47^. In fetal wounds, type III collagen is rapidly deposited in a fine reticular network that is indistinguishable from uninjured skin^48^. The healing of postnatal wounds treated with recGDNF or upon GDNF overexpression is reminiscent of fetal wound healing in terms of improved collagen distribution and HF formation when compared to controls. Importantly, fetal skin wounds develop a thickened neoepidermis^49^ compared to adult wounds, further suggesting that GDNF may stimulate early development-like features of wound repair and healing in adult mice. Hypothetically, it is possible that the improved ECM and scar maturation by GDNF may be partially due to the survival of damaged HFs and/or formation of de novo HFs following injury. These structures may be able to produce key biological growth factors that promote the formation of proregenerative cells such as adipocytes and myofibroblasts ^50^.

Although we focused on the role of GDNF signaling in hair stem cells during the wounding process, the fact that there was only partial inhibition of wound closure after *Ret* ablation suggests the presence of other cutaneous mediators of the process. Based on recent single-cell sequencing data of skin tissue^29^, *Gdnf* mRNA is present in both outer bulge stem cell compartment of hair follicles as well as epidermal cells of the interfollicular epidermis including basal keratinocytes in mice (Supplementary Fig. 7). These findings point toward the possibility that both follicular and extra follicular sources may be providing GDNF in the skin niche environment to promote wound healing after injury. Given the known regenerative functions of GDNF upon peripheral nerve injury^51^ and the fact that denervated skins in both human and animal models result in delayed wound repair^52^, it is possible that GDNF may also signal via cutaneous innervations to promote wound repair. We also observed increased vascularization upon GDNF treatment of cutaneous wounds. Recent studies also support our findings, which have shown that GDNF can stimulate capillary-like tube formation in HUVEC cells in Matrigel^53^. Furthermore, it was recently shown that GDNF may promote angiogenesis through epigenetic regulation of *FMOD* (fibromodulin)^54^, a small leucine-rich proteoglycan that is positively implicated in wound healing. Overall, a high dose of GDNF can promote hair growth and wound healing, however it is remains to be determined if GDNF signaling in other cell types besides BSCs can play a role in the natural setting.

## Methods

### Mouse strains, breeding, and husbandry

Part of the animal studies were approved by The Jackson Laboratory Animal Care and Use Committee (Permit Number: 07007) and were in accordance with the “Guide for the Care and Use of Experimental Animals” established by the National Institutes of Health (1996, revised 2011). Part of the animal studies were also approved by the Department of Veterinary Medicine, Miller School of Medicine at the University of Miami (UM) in accordance with the UM Animal Care and Use Committee (IACUC protocol #18-151; Lisse T.S.). Animals were maintained in a 12-h light and 12-h dark cycle vivarium in the Research Animal Facility at The Jackson Laboratory, as well as the animal vivarium within the Neuroscience Annex at the University of Miami. Animals were provided acidified water (pH 2.8–3.2) and autoclaved pelleted 5K52 diet (6% fat) ad libitum. In timed pregnancy crosses, pups were dated based on the presence of vaginal plugs and by noting the delivery of newly born pups. Wild-type mice used in this study consisted of C57BL/6J mice (JAX stock no. 000664; RRID: IMSR_JAX:000664). Animals carrying the *Gdnf*^*Ctsl-REB*^ transgene (i.e., founder line Tg(Ctsl-Gdnf)4352Reb, referred to as Tg(Ctsl-Gdnf) in the paper) were generated at the University of Washington and maintained on the C57BL/6J background^20^. The *Gt(ROSA)26Sor*^*tm1(CAG-Brainbow2.1)Cle*^ line (referred to as R26R-Confetti^38^; JAX stock no. 013731) was kept on the C57BL/6J strain. The *Ret*^*tm1.2Ddg*^ conditional mutant strain (referred to as *Ret*^flox 30^; JAX stock no. 028548), in which exons 14 and 15 of the mouse *Ret* gene were flanked by loxP sites, was maintained on the C57BL/6J strain. The Tg(Krt1-15-cre/PGR)22Cot line (referred to as *K15*-CrePR1^8^; JAX stock no. 005249) was on a mixed C57BL/6 and SJL background and was used for generation of RU486-induced, Cre-mediated gene deletions within BSCs of HFs. The Tg(KRT14-cre/ERT)20Efu/J line (referred to as K14-CreERT; JAX stock no. 005107) was used to generate tamoxifen-inducible gene deletions within interfollicular epidermal cells of skin. The floxed *Gfra1*^*tm2.1Jmi*^ line (*Gfra1*^EGFP^ KO allele with GFP expression; referred to as *Gfra1*^EGFP^) has been previously described^23^ and was kindly provided by Dr. Sanjay Jain (Washington University, Saint Louis). A colony of these mice was established following rederivation by in vitro fertilization of C57BL/6J oocytes and subsequently maintained on the C57BL/6J background at The Jackson Laboratory. We obtained heterozygous mice that express the *Gfra1 GFP-*knock-in allele in a ubiquitous manner by crossing *Gfra1*^*tm2.1Jmi*^ mice to *β-actin* (ACTB)-cre mice, herein referred to as *Gfra1*^EGFP/+^ or *Gfra1*^Δ/+^. The *ACTB*-Cre line (B6N.FVB*-Tmem163*^*Tg(ACTB-cre)2Mrt*^/CjDswJ; referred to as *ACTB*-Cre; JAX stock no. 019099) has been previously described^55^ and was maintained on the C57BL/6J background. For conditional deletion of *Gfra1*, the conditional-ready *Gfra1*^*tm1.1Wql*^ (Gfra1 flox) line (JAX stock no. 030611)^32^ was utilized. RU486 (M8046; 1% w/v in acetone) and tamoxifen (T5648; 20 mg/ml in corn oil, 100ul per mice; i.p.) were purchased from Sigma–Aldrich.

### Genotyping and recombination detection

For mice obtained from The Jackson Laboratory repository, genotyping was based on established protocols listed on the JAX website for each stock animal. For genotyping and quantification of Tg(Ctsl-Gdnf) transgenic mice, a PrimeTime^®^ real-time quantitative PCR multiplex assay (Integrated DNA Technologies, USA) was established with the Life Technologies ViiA7 RT-qPCR system. We used the PrimeTime^®^ Gene Expression Master Mix with low reference dye with primer and (double quench) probe solutions with 50 ng of DNA template. The PCR program was run according to the manufacturer’s recommendations (Supplementary Table 1). The *Gdnf*^*Ctsl-REB*^ transgene levels were determined by comparing delta Ct values of each unknown sample against known homozygous and hemizygous controls with an endogenous reference (Supplementary Table 1). For *Ret*^flox^ genotyping, primers Ret-F (5’-CCTTGGGCCTGCTGAGCACGGG-3′) and Ret-R (5′-GGAGGCAGGAAGGCCTGTGC-3′) were used with the PCR program: 95 °C for 3 min, and for 35 cycles 95 °C for 30 s, 66 °C for 30 s, and 72 °C for 40 s. The excised region of the *Ret* (floxed) allele after Cre-mediated recombination (null) was detected using the primers Ret-F and Ret minus-R (5′-CAAAACACTGTGCTCGCA-3′) to generate a ~500 bp amplicon using the same PCR program. For *Gfra1*^flox^ genotyping, primers Gfra1-F (5′-GGGGCGTAACTGCCAGTCAGC-3′) and Gfra1-R (5′-GTTTCAGCTCACAGGCCATTGG-3′) were used with the PCR program: 95 °C for 3 min, and for 35 cycles 95 °C for 30 s, 57 °C for 30 s, and 72 °C for 30 s. The excised region of the *Gfra1* (floxed) allele after Cre-mediated recombination (null) was detected using the primers Gfra1-F and Gfra1 minus-R (5′-GACCCAGTTCTGAGTAGGTTG-3′) to generate a 875 bp amplicon using the same PCR program but with an annealing temperature of 60 °C.

### Hair depilation, wound healing assay, and intrawound delivery of recombinant GDNF

Under general anesthesia (inhalation isoflurane), animals were subjected to depilation of dorsal hair using Wax Strips (Del Laboratories, Farmingdale, NY) or shaved to remove hair before tissue collection for analysis. All procedures were performed using sterile instruments and aseptic conditions. Experimental mice each received a full-thickness 3 mm diameter truncal wound with a sterilized biopsy punch device (Sklar Instruments, PA). Bupivacaine was provided as an analgesic and the animals were observed daily for any signs of distress. After sacrifice, the degree of re-epithelialization and tissue remodeling was monitored by harvesting tissue in 4% PFA in PBS (pH 7.4). Intrawound injections of recombinant Rat GDNF protein (50–125 µg/wound in 50 µl of vehicle solution; R&D Systems, 512-GF/CF) or 50 µl of vehicle (PBS, 1 mM KH_2_PO_4_, 155 mM NaCl, 3 mM Na_2_HPO_4_-7H_2_O, pH 7.4) was administered using a 26-gauge needle and syringe. The needle was kept in place for an additional 30 s after injection and then slowly withdrawn.

### Ultimate DISCO (uDISCO) whole-mount passive clearing technique for skin

To visualize thick skin tissue and hair morphology without sectioning, we applied the organic solvent-based uDISCO clearing method^31^. Greater than 3 mm-by-3 mm skin tissues were fixed in 4% PFA at 4 °C overnight. Samples were washed with 0.1 M PBS and subjected to a tert-butanol series (70–100%; Sigma–Aldrich, 36053) for gradient dehydration for 2–12 h. Next, dichloromethane (Sigma–Aldrich, 270997) was replaced as a pure solution for the delipidation step for 1 h at room temperature. A refractive index matching solution was prepared by mixing BABB (benzyl alcohol + benzyl benzoate 1:2; Sigma–Aldrich, 24122 and W213802) and DPE (diphenyl ether) (Sigma–Aldrich, 240834) at a BABB:DPE ratio of 10:1 (vol/vol). The samples were reacted with the BABB:DPE mixture until they became optically transparent.

### Histological and immunofluorescence analysis

Skin tissues were dissected from mice and incubated 2–4 h in 4% PFA at 4 °C before embedding in paraffin wax. 7 μm-sections were stained with hematoxylin and eosin (H&E), Masson’s trichrome and picrosirius reagents were provided by The Jackson Laboratory Histology Core Services. For some samples, cryosections were made using a Leica CM 1950 Cryostat. For immunofluorescence studies, sections were deparaffinized in xylene and rehydrated, the subjected to antigen retrieval for certain antigens (e.g., α-Ki67). Tissue sections were blocked in PBS containing 3% normal goat serum and then incubated with primary antibodies overnight at 4 °C. Primary antibodies used in this study included: Ki67 (Abcam 16667), KRT15 (Abcam 52816; RRID:AB_869863), RET (Santa Cruz Biotech. Sc167; RRID:AB_631317), GFRA1 (Santa Cruz Biotech. Sc10716; RRID:AB_2110298), CD31 (Abcam 28364), COL4a1 (Abcam 19808), and GDNF (Santa Cruz Biotech. Sc328) at 1:200 dilution. Following 3x washes for 5 min each in PBS, the sections were incubated at room temperature for an hour with corresponding species-specific secondary antibodies (Alexa series, Life Technologies). Following 3× washes in PBS in the dark, sections were mounted with Vectashield medium containing DAPI (Vector Laboratories) for nuclei staining. Laser scanning confocal microscopy was performed using an inverted Olympus FV1000 unit. *Z*-stacks were obtained from 1–3 µm/slice at a speed of 12.5µs/pixel and then maximally projected. All other parameters (e.g., pinhole diameter, gain, laser intensities) were kept constant. Using the FluoView FV1000 imaging software (Olympus), we obtained orthogonal regions (XY, XZ, or YZ) by placing a crosshair at a point of interest to obtain a plane along the crosshair lines.

### Lineage tracing of hair follicle stem cells after injury with the R26R-Confetti reporter line

For *K15*-CrePR1:R26R-Confetti sample preparation, dorsal hair was removed with a shaver and wounded skins (2 × 2 cm^2^) were fixed in 4% PFA in PBS (pH 7.4) for 2 h. Skin and hair-containing tissues derived from wounds were cut along the midline and then subjected to graded sucrose treatments (15–30%) for cryoprotection. These tissues were embedded face down along the midline in OCT embedding medium (Histolab). Four serial sections per wound sample were collected and stained with the DRAQ5™ (Thermo Scientific) far-red DNA fluorescent probe to define cutaneous structures such as the neoepidermal boundaries. Sections were then cover slipped using DAPI-less Vectashield anti-fade mounting medium (Vector Laboratories) for confocal imaging. Imaging was performed using an LSM SP8 inverted confocal microscope (Leica) using a 10 × /0.4 multi-immersion objective. The Confetti fluorescent proteins were detected using the 405 nm (CFP), 488 nm (GFP), 514 nm (YFP) and 561 nm (RFP) lasers, and the images were acquired at 1024 × 1024 resolution. The 681 nm (DRAQ5) laser channel was also established, and the super-sensitive HyD detector was used for low-emitting CFP detection. Initially, laser power and gain were adjusted manually to provide optimal fluorescence intensity with minimal photo-bleaching. The step size (1) and line averaging (4) were kept the same for all samples. Projection of z-stack planes and image analysis were performed using the IMARIS (Bitplane) software. For the Confetti lineage tracing parameters, all calculations were based on events which occurred within the neoepidermal borders between the wound edges. A single “Confetti” clone was defined as cohesively associated cells of the same color, whereby the number of cohesively associated cells was the clone size. The number of individual fluorescent colors (termed XFPs) was also calculated, whereby the maximal possibilities are four per wound section. All plots were generated in Prism 3 (Graphpad).

### Bulge stem cell isolation and colony formation assay

In all, five male C57Bl/6J mice of 53 days of age were used to harvest BSCs. Dorsal skins were placed on a sylgard plate, cleared of fat, and then incubated at 37 °C for 120 min with 0.25% trypsin (Gibco). The hair follicles were scrapped from the skin and transferred to a new culture dish to prepare a single-cell suspension. The suspension was washed with staining buffer consisting of 3% Chelexed (Chelex^®^-100; BioRad) FBS in PBS without Ca^2+^ and Mg^2+^ and kept on ice. The cells were then passed through a 70 µm cell strainer and then a 40 µm cell strainer. 12.5 × 10^6^ cells were recovered (2.5 × 10^6^/mouse), which were subjected to cell sorting. Spectral separation of the conjugated antibodies was confirmed using the BD website, and the antibodies were added to the cells at 1:100 (CD34-FITC; 11-0341-82, Life Technologies) and 1:50 (CD49f-PE/Cyanine7; 313621, BioLegend) in staining buffer. Mouse BSCs were isolated using a BD FACSAria II cytometer and analyzed using FACSDiva (ver. 8.0.2) at the Sylvester Comprehensive Cancer Center (University of Miami Miller School of Medicine). Appropriate controls were included, and a post-sort analysis was also performed (Supplementary Fig. 8). In all, 2.5 × 10^6^ BSCs were purified from the starting material. For colony formation assays, approximately 1000 BSCs/cm^2^ were seeded onto collagen-coated (ALX-522-440-0050; Enzo Life Sciences) plates along with mitomycin-treated NIH/3T3 (CRL-1658; ATCC) feeder cells in Dulbecco’s Modified Eagle’s Medium (30-2002; ATCC) with 10% fetal calf serum. BSCs were analyzed in triplicate and treated with carrier-free recombinant GDNF (01-A0460; ORF Genetics) reconstituted in sterile PBS. Colonies were imaged on days 3 and 6 using a PrimoVert inverted microscope (Zeiss), and the media was replaced on day 3. Colony size was determined using ImageJ and the data was analyzed using Prism (Graphpad).

### RNA isolation and evaluation of gene expression

Total RNA was extracted using the RNeasy Plus minikit (QIAGEN) according to the manufacturer’s recommendations. Total RNA (1 µg) was reversed transcribed using the Superscript III first-strand synthesis system (Life Technologies) with random hexamers following the manufacturer’s recommended conditions. Quantitative real-time PCR (qPCR) was performed using primers designed to span introns based on the latest genome assembly, GRCm38.p5 (Supplemental Table 2), using the SYBR Green PCR Master Mix (Applied Biosystems). Measurements were made on a Life Technologies ViiA7 RT-qPCR system in a 384-well format, and cycling was followed by melt curve analysis to identify spurious amplification. Target gene expression was normalized to beta-actin mRNA in the same sample, and relative gene expression was calculated using the method of Livak and Schmittgen. Statistics were performed using Prism 7 (GraphPad Software) and analyzed with an unpaired Student’s *t*-test (error bars are reported as SEM).

### Data analysis

For histomorphometry of homozygous Tg(Ctsl-GDNF) HFs, three serial sections were used for quantifying the number of anagen HFs present within a section imaged at ×4 magnification (i.e., equaling 2 mm). For histomorphometry of P40 *ACTB*-Cre:*Ret*^Δ/+^ mice, each experimental animal skin was processed for H&E staining and quantified using three serial sections followed by an 100 μm interval and repeated three times in all (nine sections per animal). The total number of HFs was appraised using a ×4 objective (i.e., to obtain a 2 mm field of view) of a brightfield microscope, whereby the data were then converted into percentages of remaining HFs in the hypodermis. For histomorphometric analysis of *K15*-Cre:*Ret*^flox/flox^ mice, four serial sections of skin samples (i.e., to appraise post depilation and the hair cycle at P29) were used to quantify the number of anagen IV-VI stage HFs present. We used the ×4 objective for the analysis, thereby the values represent the average number of follicles present within a 2 mm section of skin. For *K14*-Cre:*Ret*^flox/flox^ and *K15*-Cre:*Gfra1*^flox/flox^ skin thickness determination, 4–6 serial sections of skin per mice were analyzed by taking repeated measurements across the span of skin in a blinded fashion.

### Reporting summary

Further information on research design is available in the Nature Research Reporting Summary linked to this article.

## Supplementary information

Supplemental Materials

Reporting Summary

## Data Availability

All data generated during and/or analyzed during the current study are available from the corresponding author on reasonable request.
