# Supplementary material for: GDNF promotes hair formation and cutaneous wound healing by targeting bulge stem cells
Source: NPJ Regen Med. 2020 Jun 12;5:13. doi: 10.1038/s41536-020-0098-z (PMC7293257; doi:10.1038/s41536-020-0098-z)
Supplement: Supplementary file 1 — Supplemental Materials [file 41536_2020_98_MOESM1_ESM.pdf]

Supplemental Information

GDNF promotes hair formation and cutaneous wound healing by targeting bulge stem cells

Thomas S. Lisse[1,2,3†*], Manju Sharma[1†], Neda Vishlaghi[2], Sri Ramulu Pullagura[1,4] and Robert E. Braun[1,4*]

[1] The Jackson Laboratory, 600 Main Street, Bar Harbor, Maine 04609 USA
[2] The University of Miami, Department of Biology, Coral Gables, FL 33124 USA
[3] Sylvester Comprehensive Cancer Center, Miller School of Medicine, University of Miami, Miami, FL 33146 USA
[4] Graduate School of Biomedical Sciences and Engineering, University of Maine, Orono, 04469 Maine USA

[†] Contributed equally to the manuscript
* Corresponding authors

Dr. Robert Braun, 207-288-6841, bob.braun@jax.org
Dr. Thomas Lisse, 305-284-3957, tom.lisse@miami.edu

Keywords: hair follicle, hair cycle, bulge stem cell, wound, wound repair, wound healing, regeneration, hair regeneration, neurotrophic factor, neurodegeneration, neuron, nerve cells, brain, Gdnf, Ret, Gfra1, growth factors

Supplemental Figures

Figure S1.

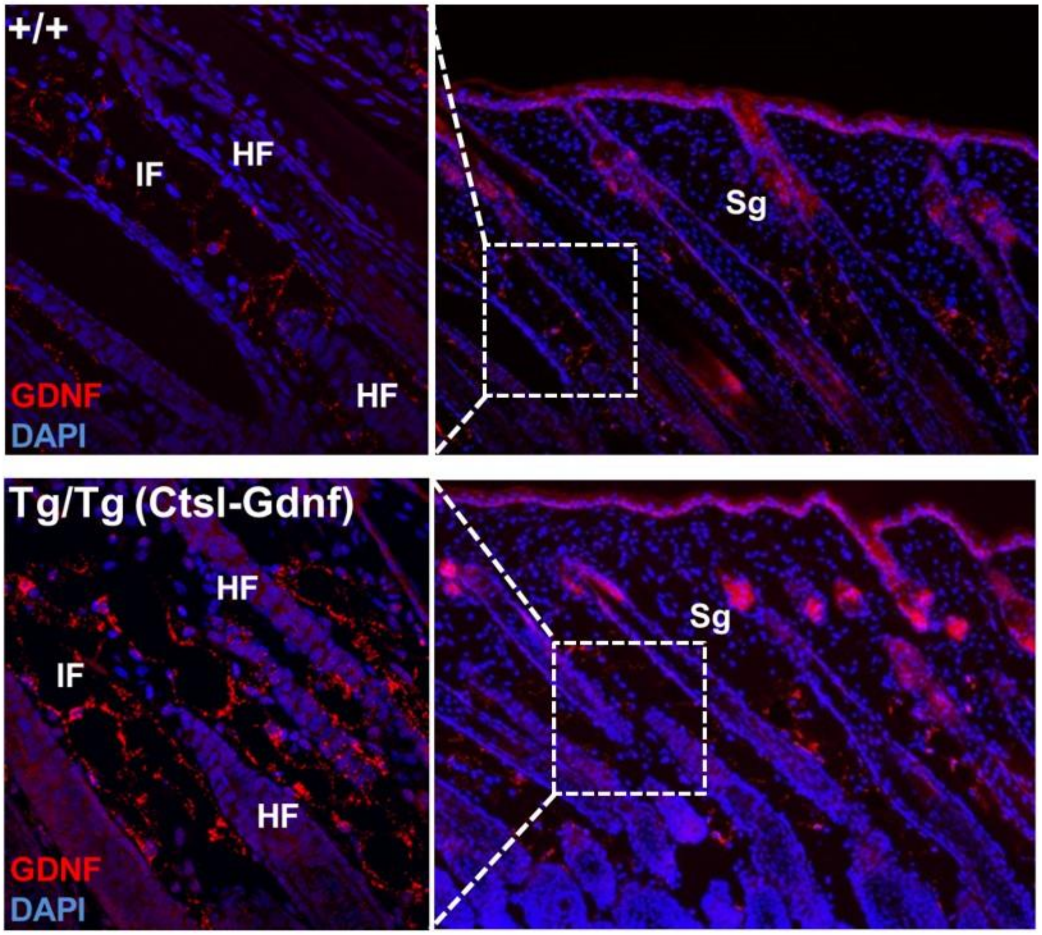

Figure S1. Transgenic mouse model with overexpression of GDNF in the skin. Related to Figure 1c.

Immunofluorescence analysis shows increased GDNF expression in homozygous Tg(Ctsl-GDNF) skin within the interfollicular space and sebaceous glands (Sg) relative to non-transgenic animals (+/+). Hair follicle (HF), interfollicular space (IF)

Figure S2.

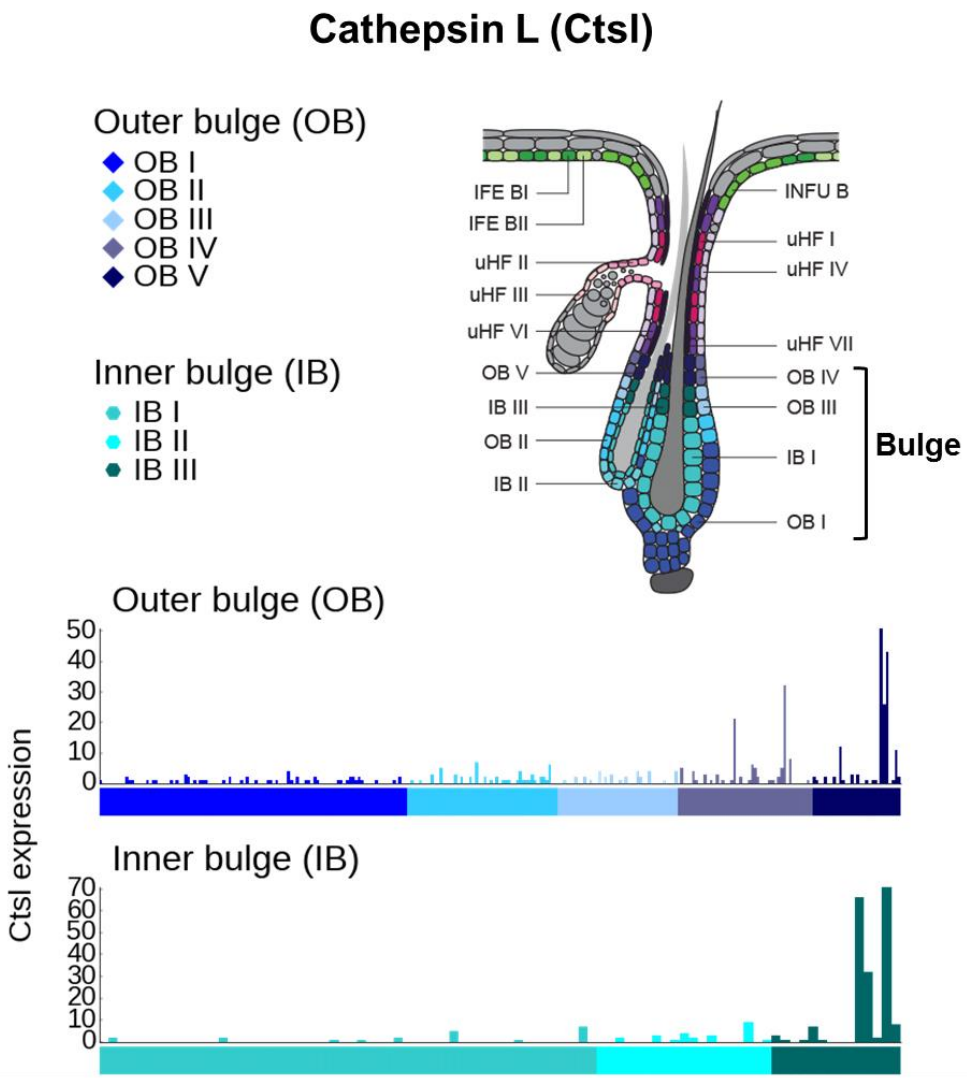

Figure S2. ***Ctsl*** **is enriched in hair bulge stem cells of hair follicles. Related to Figure 1c.**

Within the hair follicle, single cell transcriptomic analysis revealed that *Ctsl* is expressed within the outer bulge V and inner bulge III layers closest to the sebaceous glands. Data obtained from Joost et al. 2016 (Joost et al., 2016), which was published under the terms of the Creative Commons Attribution-Non Commercial-No Derivatives License (CC BY NC ND), which can be used without permission from Elsevier.

Figure S3.

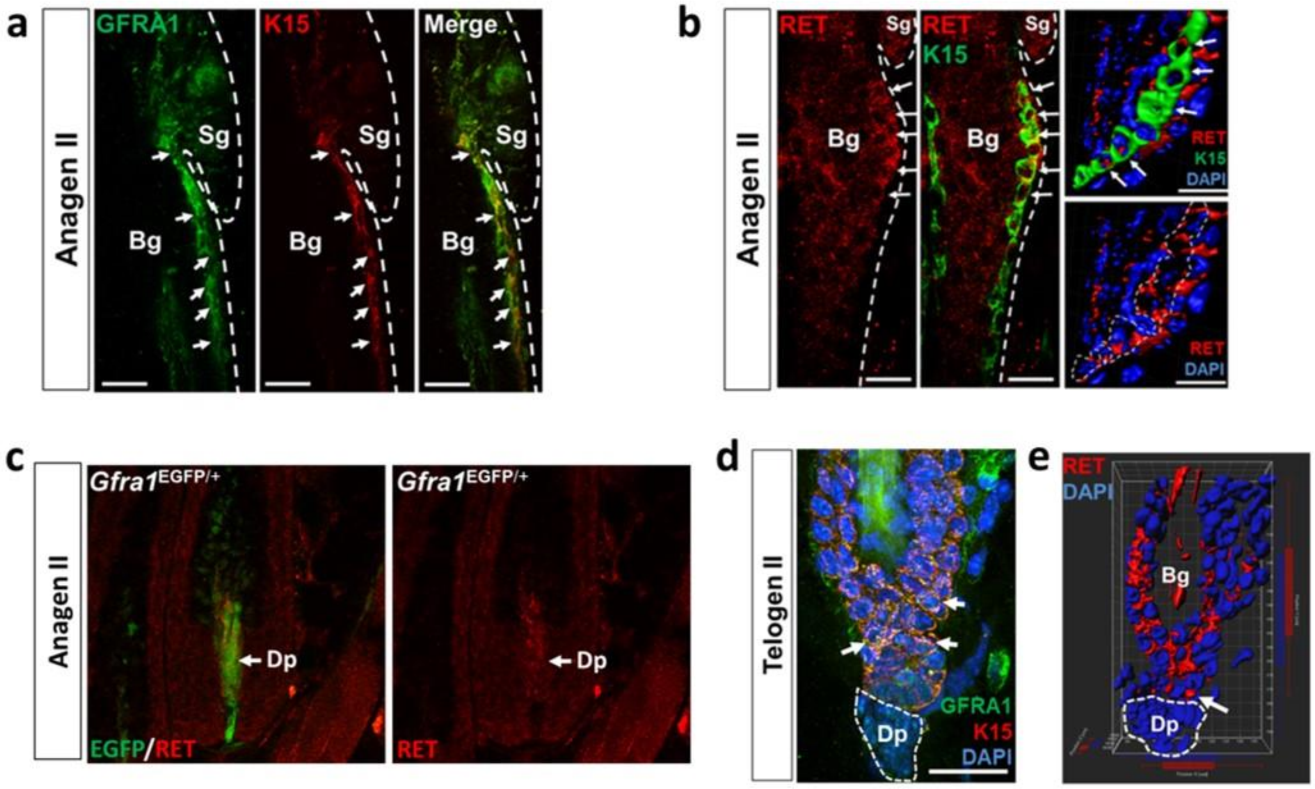

Figure S3. Antibody labeling of GDNF-responding cells in hair follicles. Related to Figure 3.

a) GDNF targets a subpopulation of GFRA1$^+$/K15$^+$ BSCs during anagen II. K15 and GFRA1 co-labeled cells (white arrows) in the bulge (Bg) region below the sebaceous (Sg) glands of HFs (outlined in white). Bars = 20µm

b) GDNF targets a subpopulation of RET$^+$/K15$^+$ BSCs during anagen. K15 and RET co-labeled cells (white arrows) in the bulge (Bg) region below the sebaceous glands (Sg) of the HF (outlined in white). 3D-isosurfacing (Imaris) depicted an intracellular localization pattern within individual BSCs (left panels). Bars = 20µm

c) RET is expressed within GFRA1-EGFP^{+} dermal papillary (Dp) cells of anagen hair follicles. Co-localization studies using *Gfra1*$^{EGFP/+}$ mice shows RET immunofluorescence expression within GDNF-responding Dp cells in anagen hair follicles.

d) GDNF targets a subpopulation of GFRA1^{+}/K15^{+} BSCs during telogen II. Presence of GFRA1-K15-positive BSCs (white arrows) depicting a yellow overlay signifying co-localization. Dermal papilla (Dp). Bar = 20μm

e) Presence of RET^{+} cells within the bulge compartment of telogen HFs. 3D-isosurfacing of RET shows intracellular localization within cells of the bulge (Bg) compartment. Hair germ cell adjacent the dermal papilla (white arrow) depicts intracellular sequestration of RET.

Figure S4.

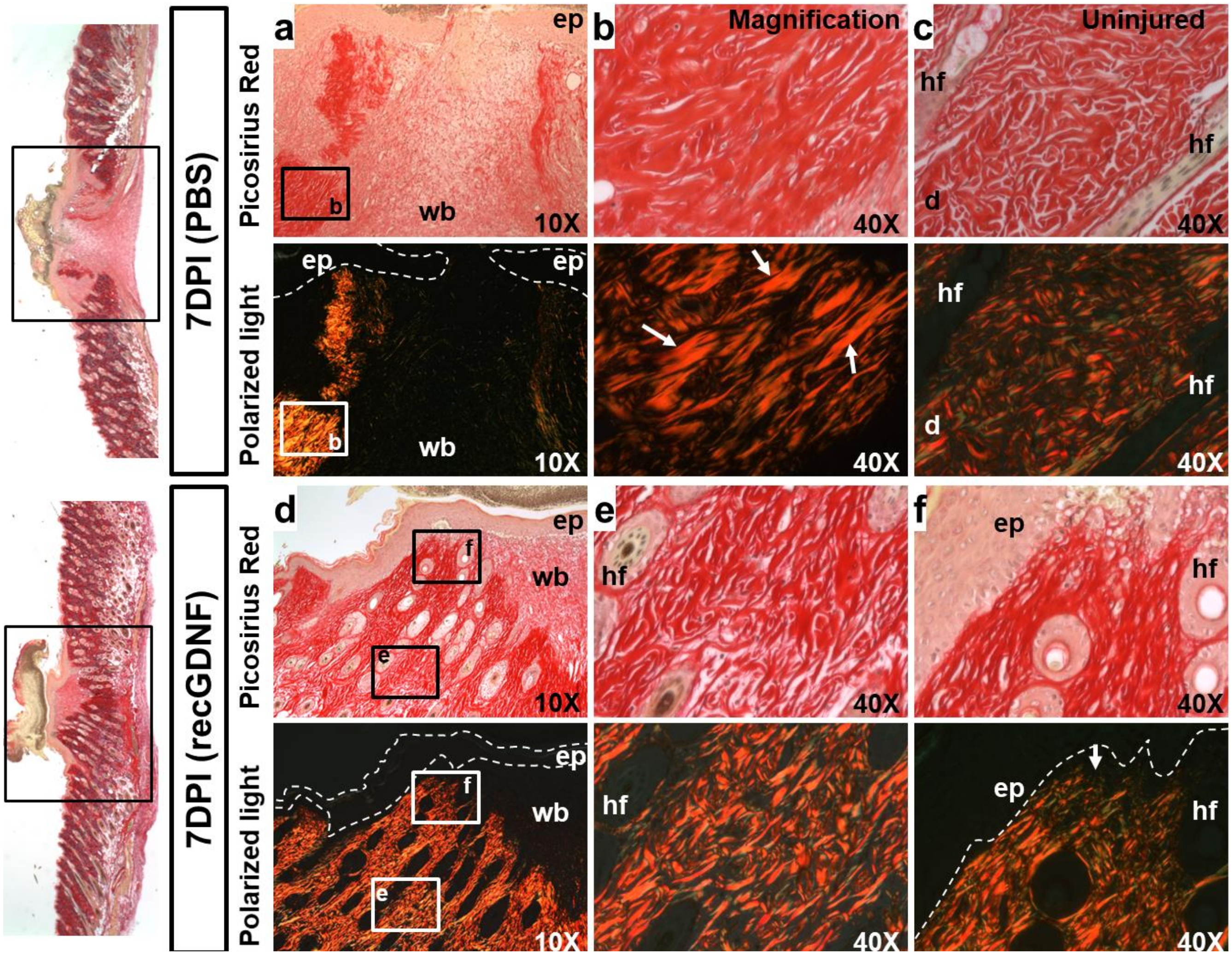

Figure S4. Recombinant GDNF treatment improves collagen organization and maturation during wound repair. Related to Figure 6d-e.

a-c) Vehicle (PBS)-treated wounds at 7 days post injury (DPI). Picosirius red stained sections are in the upper panel, and the polarized light images are in the lower panels. An entire section is depicted in the

left panel. Panel a: section depicting the wound bed (Wb). Box region is magnified in panel B. Panel c: section of uninjured representative tissue. In the lower panel b, arrows depict thick bundles of deposited collagen organized in parallel orientation.

d-f) Recombinant GDNF (recGDNF)-treated wounds at 7 DPI. Panel d: Entire wound bed region is depicted. Panel e: magnification of wound bed region 1. Panel f: magnification of wound bed region 2. Arrow depicts region of intense green birefringence, marking woven collagen. Epidermis (ep), dermis (d), hair follicle (hf)

Figure S5.

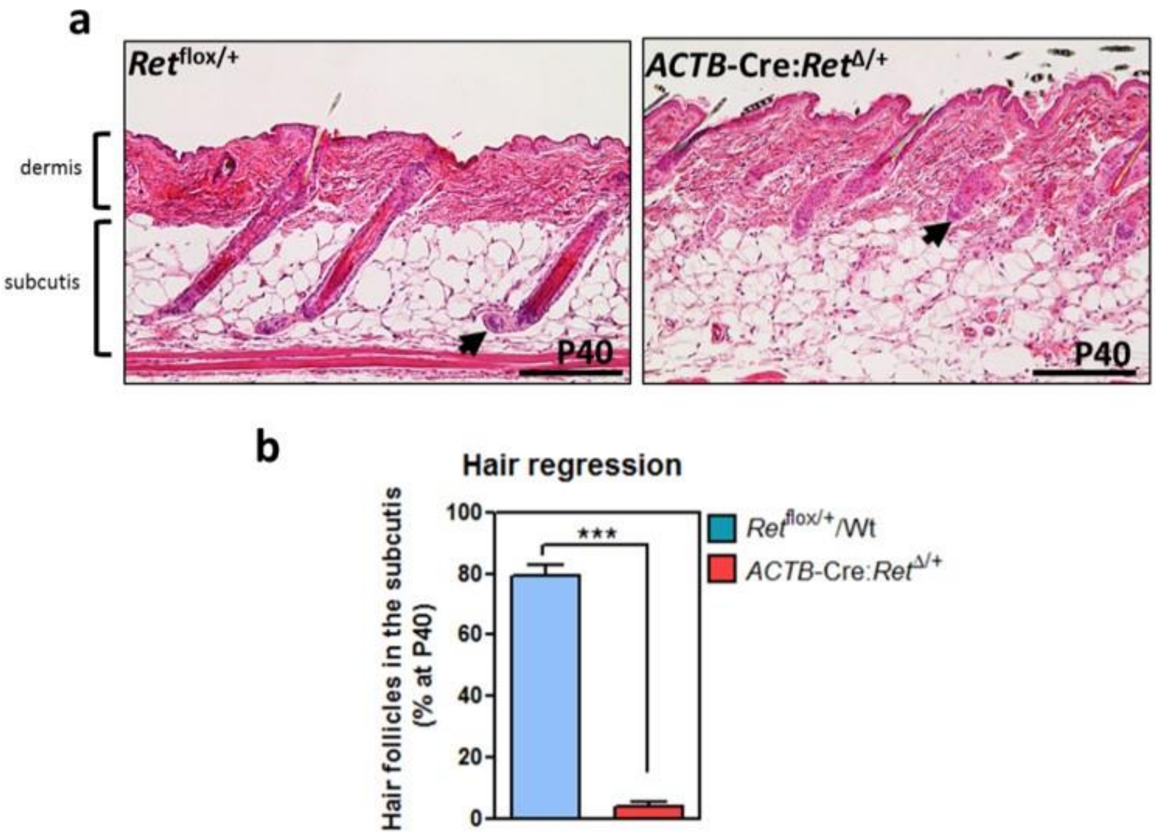

Figure S5. Global *Ret* haploinsufficiency enhances hair follicle regression. Related to Figure 8.

a) Comparison between control ($Ret^{flox/+}$) and mutant ($ACTB$-Cre:$Ret^{\Delta/+}$) stage of the hair cycle at P40. Arrow in $ACTB$-Cre:$Ret^{\Delta/+}$ skin depicts the dermal papilla (Dp) of a HF, which has retracted up into the dermis. In contrast, control follicles still contain the epithelial strand (including the Dp) still within the subcutis of the skin layer. Bar = 200µm

b) Percentage of HFs remaining in the subcutis of skin at P40. n=7, control $Ret^{flox/+}$ and $Ret^{+/+}$ male mice; n=6, mutant $ACTB$-Cre:$Ret^{\Delta/+}$ male mice; ***p<0.0001; error bars = SEM, Student’s two-tailed t test.

Figure S6.

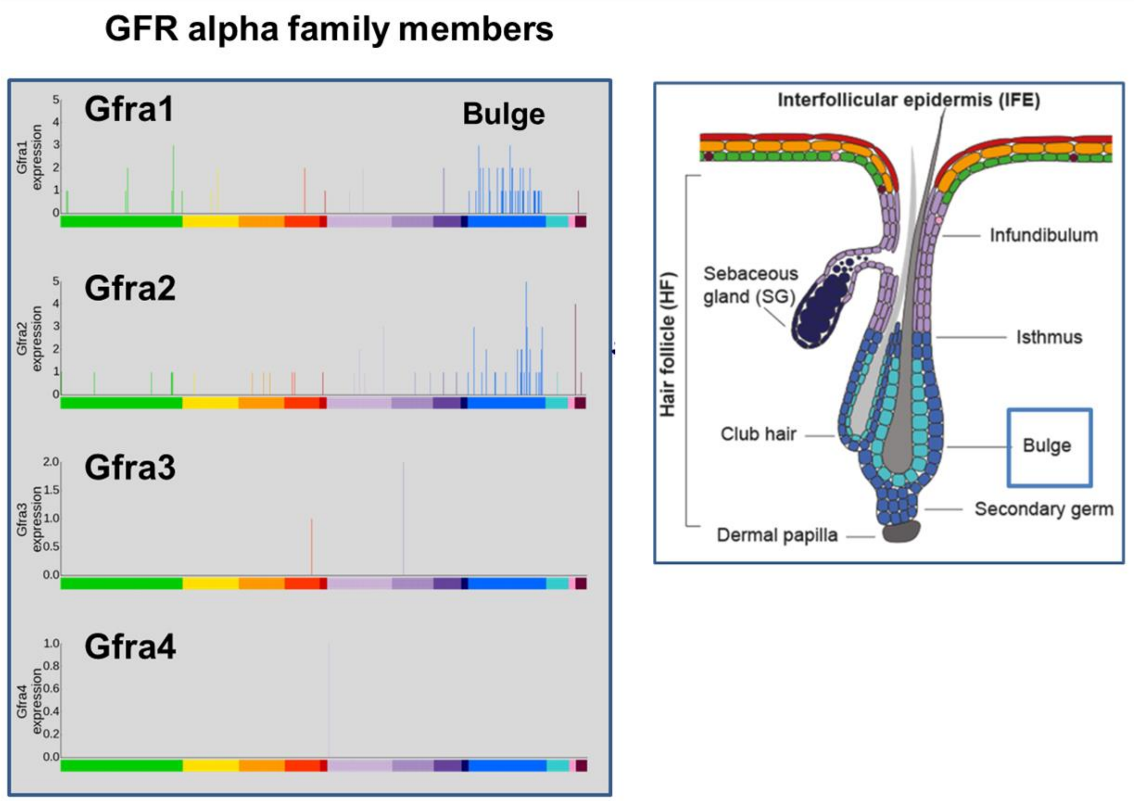

Figure S6. GFRα family members 1 and 2 are enriched in hair bulge stem cells within resting hair follicles.

Single cell transcriptomic analysis of skin cells revealed that resting hair follicle BSCs are enriched for GFRα1 and 2. The natural ligand for GFRα2 is neurturin, while GDNF is the natural ligand for GFRα1. Data obtained from Joost et al. 2016 Cell Systems.

Figure S7.

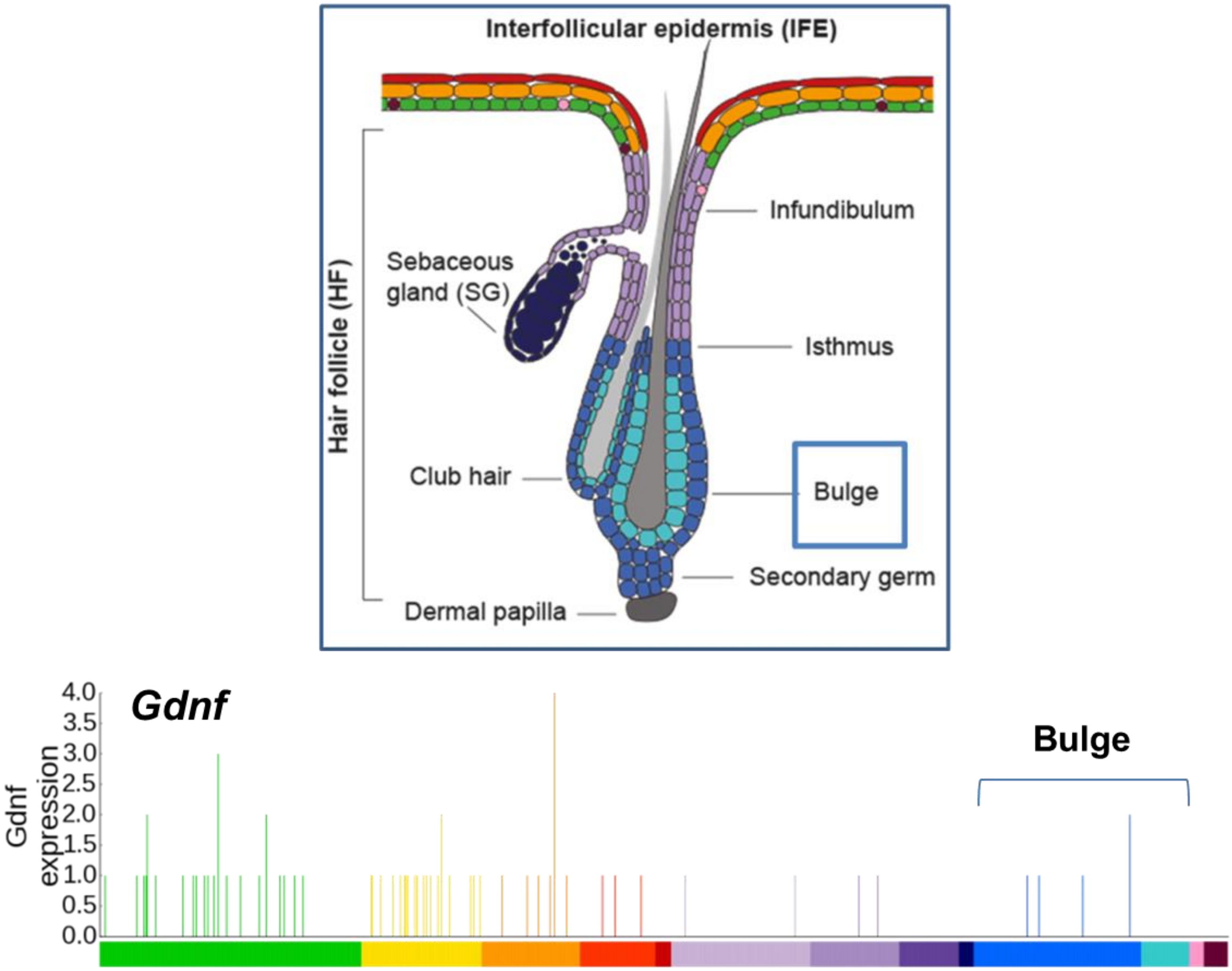

Figure S7. ***Gdnf*** **mRNA expression in single cells of mouse skin. Related to Figures 6 and 8.**

Single cell sequencing results from mouse skin reveal that Gdnf transcripts are present in both bulge hair follicle stem cells, as well as keratinocytes of the interfollicular epidermis (Joost et al. 2016), suggesting a diverse population of cells which can influence wound healing responses.

Figure S8.

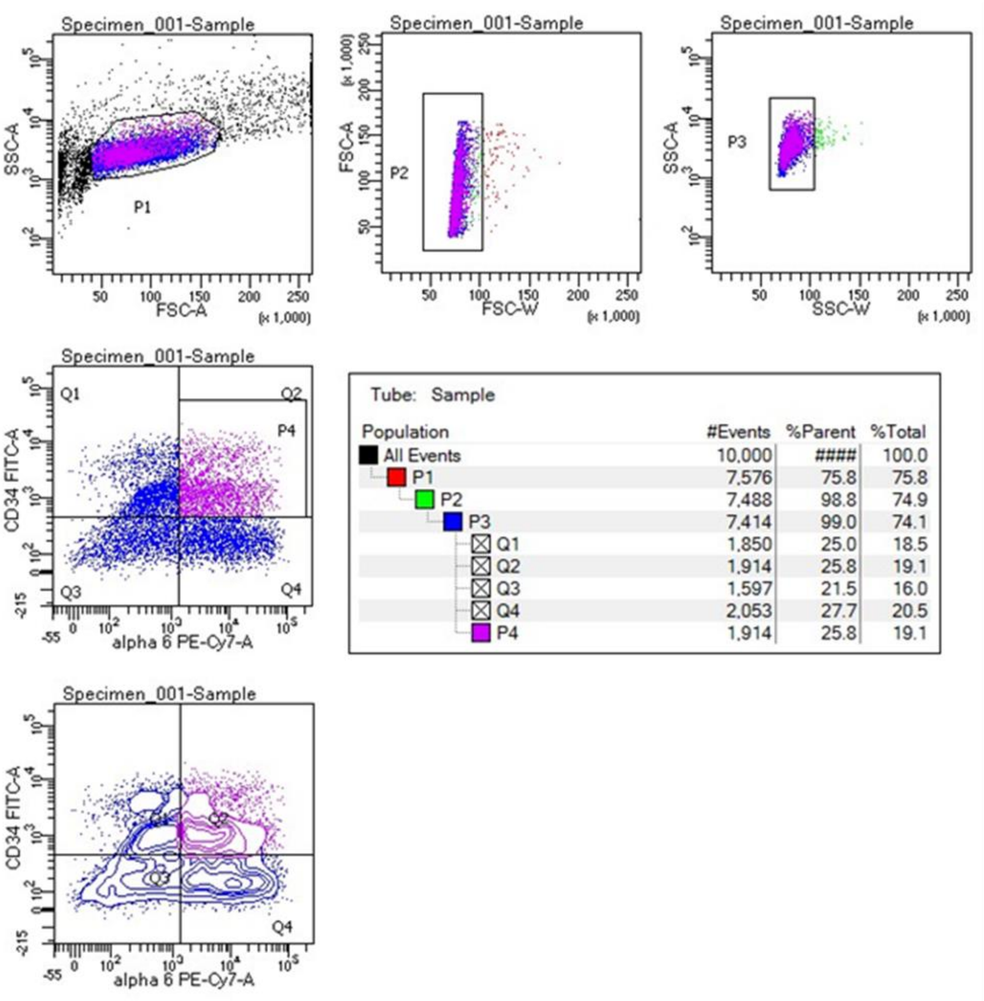

Figure S8. Gating strategy for purification of BSCs.

Supplementary Tables

Supplementary Table 1. Genotyping Tg(Ctsl-Gdnf)4352Reb transgenic mice and quantification of the *Gdnf*$^{Ctsl\text{-}REB}$ transgene

Tg(Ctsl-Gdnf) genotype				
Primer type	5’-3’ sequence	GC content (%)	Tm °C (50mM NaCl)	Reference
Internal control – Forward	CACGTGGGCTCCAGCATT	61	58	NM_009693.2
Internal control - Reverse	TCACCAGTCATTTCTGCCTTTG	46	60	NM_009693.2
Internal control – Probe	Cy5-CCAATGGTCGGGCACTGCTCA-Iowa Black® RQ-Sp	62	65	NM_009693.2
GFP transgene – Forward	AGCAGAAGAACGGCATCAA	47	55	GenBank: L29345.1
GFP transgene – Reverse	GTGCTCAGGTAGTGGTTGTC	55	55	GenBank: L29345.1
GFP transgene – Double quench probe	6-FAM™-CAAGATCCG-ZEN-CCACAACATCGAGGA-Iowa Black® FQ	54	61.1	GenBank: L29345.1

Supplementary Table 2. Real-time PCR primer set (mouse)

Target	Forward (5’-3’)	Reverse (5’-3’)	Reference
Krt40	TGCCAGACTGAGATGTTGGA	GCCCCTGTACGTGTTGATCT	NM_001039666
Bmp4	CGTCATTCCGGATTACATGA	GCTGCTGAGGTTGAAGAGGA	NM_007554
Bmpr1a	CGGTACATGGCTCCAGAAGT	ACACACAACCTCACGCATGT	NM_009758
Bmpr2	TGGCAGTGAGGTCACTCAAG	GATTTTTGGCACACGCCTAT	NM_007561
Wnt10b	CGAGAATGCGGATCCACAA	CCGGTTCAGGTTTTCCGTTA	NM_011718
Dkk1	ATGAGGCACGCTATGTGCT	TCTGATGATCGGAGGCAGAC	NM_010051
Dkk2	GCATAGAGATCGCAACCATGGTCAC	CTGATGGAGCACTGGTTTGCAGAT	NM_020265
Ret	TGGCAATTGAGTCCCTTTTC	AGCAAACACTGGCCTCTTGT	NM_001080780

B-actin	GTCAGGATACCTCTCTTGCTCTG	CCAGTTCGCCATGGATGACGATAT	NM_007393
